# Non-antifungal medications administered during fungal infections drive drug tolerance and resistance in Candida albicans

**DOI:** 10.1099/jmm.0.002046

**Published:** 2025-07-28

**Authors:** Mariella Obermeier, M. Alejandra Esparza-Mora, Olivia Heese, Nir Cohen, Sreejith Jayasree Varma, Pinkus Tober-Lau, Johannes Hartl, Florian Kurth, Judith Berman, Markus Ralser

**Affiliations:** 1 Department of Biochemistry, Charité – Universitätsmedizin Berlin, Berlin, Germany; 2 Department of Infectious Diseases and Critical Care Medicine, Charité – Universitätsmedizin Berlin, Berlin, Germany; 3 Berlin Institute of Health at Charité – Universitätsmedizin Berlin, Berlin, Germany; 4 Shmunis School of Biomedical and Cancer Research, George S. Wise Faculty of Life Sciences, Tel Aviv University, Ramat Aviv, Israel; 5 Max Planck Institute for Molecular Genetics, Berlin, Germany; 6 Centre for Human Genetics, Nuffield Department of Medicine, University of Oxford, Oxford, UK

**Keywords:** antifungal resistance, antifungal tolerance, *Candida albicans*, drug interactions, fungal infections, treatment failure

## Abstract

**Introduction.** Fungal infections are increasingly concerning, particularly in immunocompromised patients. These patients often suffer from comorbidities and receive multiple, non-antifungal medications.

**Gap Statement.** The effects of these co-administered medications on fungal cells – and their potential to influence antifungal drug efficacy – are poorly understood.

**Aim.** This study investigates non-antifungal medications commonly administered in parallel to antifungals and evaluates their impact on fungal susceptibility.

**Methodology.** We systematically reviewed clinical guidelines to identify non-antifungal medications frequently co-prescribed with antifungals. Focusing on *Candida albicans*, the most prevalent fungal pathogen, we examined whether the presence of these drugs influences antifungal responses of *C. albicans*. First, we tested the selected compounds together with antifungals in combination assays. Interactions were then characterized using checkerboard assays, and the impact on antifungal resistance and tolerance was evaluated through disc diffusion assays. To further explore these effects *in vivo*, the influence of selected antagonistic interactions on treatment efficacy was assessed using a *Galleria mellonella* model of disseminated candidiasis.

**Results.** From 119 medications used to manage 40 conditions linked to a high risk of fungal infections, we identified 34 compounds that altered the effectiveness of the antifungals fluconazole (FLC) and/or anidulafungin. Most of these compounds reduced or antagonized antifungal efficacy, often due to increased resistance or tolerance. Validation in a *G. mellonella* infection model confirmed that compounds antagonistic to FLC, including loperamide, estradiol and levothyroxine, interfere with antifungal treatment efficacy in this *in vivo* model.

**Conclusion.** Our findings highlight that medications frequently used by patients at risk for fungal infections can inadvertently increase fungal pathogen drug tolerance or resistance. We suggest that drugs targeting non-fungal conditions yet affecting fungal pathogens might represent an underestimated factor contributing to rising antifungal resistance and tolerance.

## Data Summary

All hits of the systematic guideline search as well as the composition of the drug shortlist are described in Supplementary Tables. Complete results of the targeted drug testing, checkerboard assays, disc diffusion assays and *Galleria mellonella* experiments are shown in Supplementary Figures and Supplementary Tables.

## Introduction

Fungal infections represent a substantial medical challenge, affecting more than one billion people globally [1]. The infections range from mild skin or nail conditions to irritating mucosal or vaginal infections and to an increasing number of life-threatening invasive diseases. Recognizing the urgent need to address the global health burden of these infections, the World Health Organization released the first Fungal Priority Pathogens List in 2022, ranking the ascomycete yeast *Candida albicans* in the highest category of critical pathogens [2]. Indeed, *C. albicans* is a leading cause of mortality in intensive care units, and up to 40% of the most severe invasive infections fail to respond to antifungal treatment [3,4]. Resistance against antifungals is a known but still minor contributor to these treatment failures. However, a related phenomenon, antifungal tolerance, in which subpopulations of cells maintain growth above the minimal inhibitory concentration (MIC) is commonly observed in clinical isolates and seems to be rising rapidly [5].

Some individuals are particularly at risk for a life-threatening progression of fungal infections. These include patients with immunosuppression, those who have undergone abdominal surgery, those in intensive care units and those with severe wounds [3,6, 7]. Many of these conditions require complex medical regimens. The resulting polypharmacy can trigger drug–drug interactions (DDIs) that affect the efficacy of the antifungal drugs [8]. For example, an antagonism between the azole antifungal fluconazole (FLC) and the antibacterial sulfadiazine increased the growth of *C. albicans* cells present in a liquid culture compared with the culture containing only FLC [9].

Despite the recognition that DDIs induced by drugs directed against non-fungal diseases can alter antifungal responses in the pathogen, only a limited number have studied the role of DDIs in the context of antifungal responses systematically, and these mostly focussed on the possibility of drug repurposing rather than antagonism [10,13]. Moreover, there is limited data available on ‘drugs of clinical relevance’ that are frequently administered to patients who suffer from fungal infection as a comorbidity or as a treatment side effect. To address this problem, we mined the medical guidelines collected by the Association of the Scientific Medical Societies (AWMF) in Germany [14], for references to fungal infection comorbidities alongside a wide range of diseases. We used the guidelines to identify and to prioritize the most commonly administered medications co-administered next to the antifungals, to patients with fungal infections or risk of fungal infections. We then tested 119 shortlisted compounds in combination with standard antifungals in *C. albicans* cultures. We report multiple DDIs with the antifungals FLC and anidulafungin (ANI), two common antifungals used to treat *C. albicans* infections. While a small number of the tested drugs increased the antifungal efficacy, we obtained a higher hit rate of compounds that negatively influenced the efficacy of the antifungal. This included several interactions which enhanced drug resistance and/or tolerance of the pathogen *in vitro* and reduced antifungal treatment efficacy in a simple *in vivo* model for invasive *candidiasis*.

## Methods

### Culture conditions

All experiments were performed using *C. albicans* SC5314, a strain initially isolated from a patient diagnosed with candidiasis in the early 1980s, which currently serves as a global reference in laboratories doing research on *C. albicans* [15]. Fungal cells were kept at −80 °C in 20% glycerol YPB (yeast extract peptone dextrose medium) stocks. Two days before the experimental start, *C. albicans* was streaked onto YPD agar plates and incubated at 30 °C with 60% humidity. After 36 h of incubation, three colonies were selected and inoculated in 10 ml YPD (drug screen and checkerboard assays), followed by overnight (ON) cultivation (30 °C, 60% humidity, shaking). Notably, in disc diffusion experiments, three colonies were individually inoculated for the ON culture. The next day, the culture was diluted 1:40 in synthetic minimal medium (SM). One litre SM was prepared from 6.7 g yeast nitrogen base without aa (291940, Thermo Fisher Scientific) and 2% glucose, buffered to pH 6.55 using MOPS and filled up with sterile mq H_2_O. Before starting an experiment, the pre-culture was incubated for 4 h (30 °C, 60% humidity, shaking).

### Targeted drug testing

**Fig. 1.**
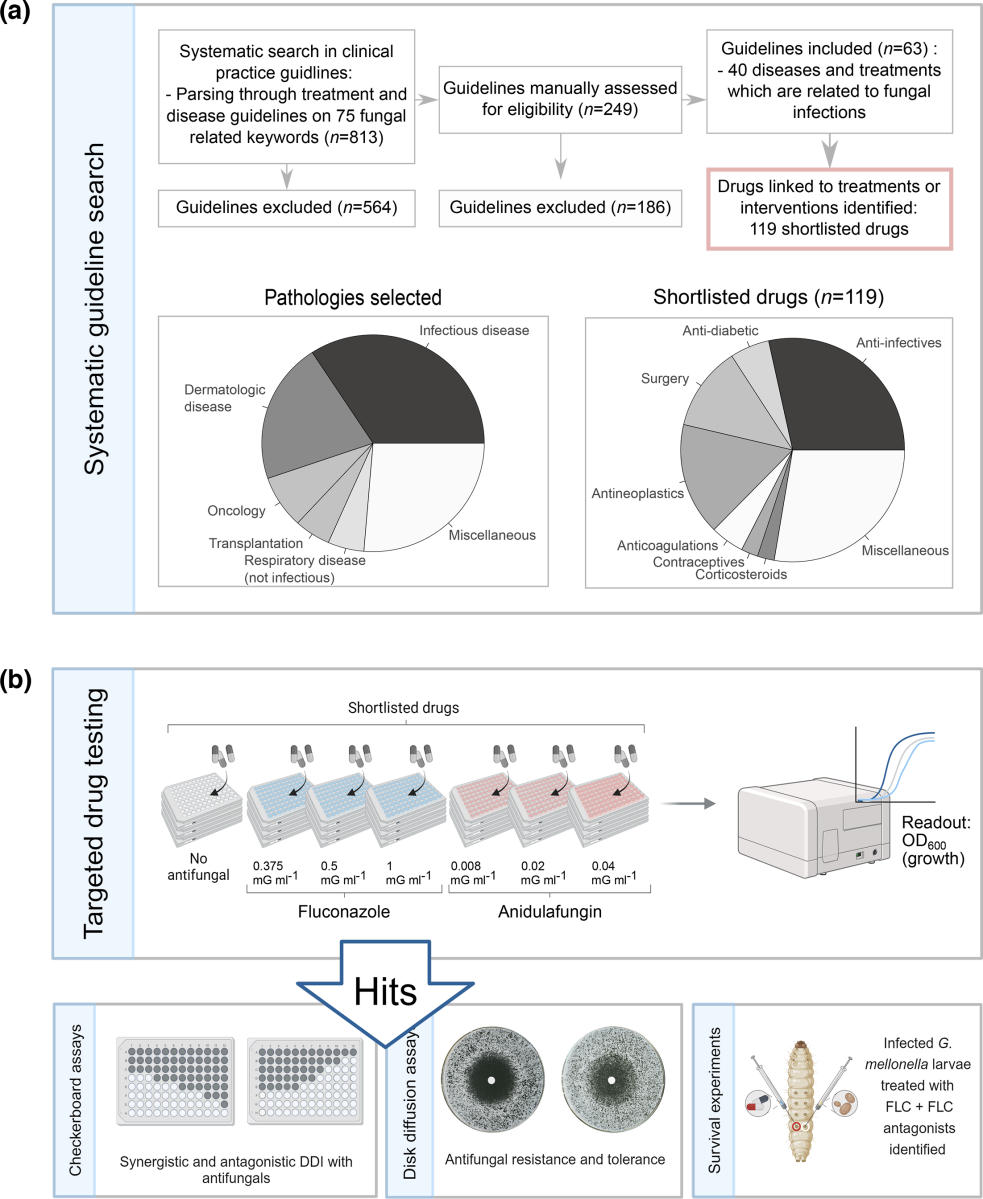
Workflow of the medical guideline search and experimental set-up. (**a**) To identify pathologies in fungal infection high-risk settings, 813 medical guidelines were systematically mined for their association with fungal infections. The search resulted in 63 guidelines which indicate a relation of 40 pathologies or medical interventions to fungal infections. A total of 119 compounds directed against non-fungal diseases which are commonly used in these identified pathologies and interventions were shortlisted. Pathology classes selected in the systematic keyword search and related shortlisted drugs are shown in the pie charts. (**b**) While targeted drug testing, the shortlisted drugs were systematically exposed to *C. albicans* SC5314 cultures in the presence and absence of the antifungals FLC and ANI. Hits, defined as compounds which increased or decreased the OD_600_ after 60-h culturing, were further tested in checkerboard assays to identify synergistic and antagonistic DDIs, and DDAs to determine whether the interactions altered antifungal tolerance and/or resistance. Finally, FLC antagonists identified from checkerboard assays were co-administered with FLC treatment in *G. mellonella* larvae infected with *C. albicans*. Larval survival was tested every 24 h.

**Fig. 2.**
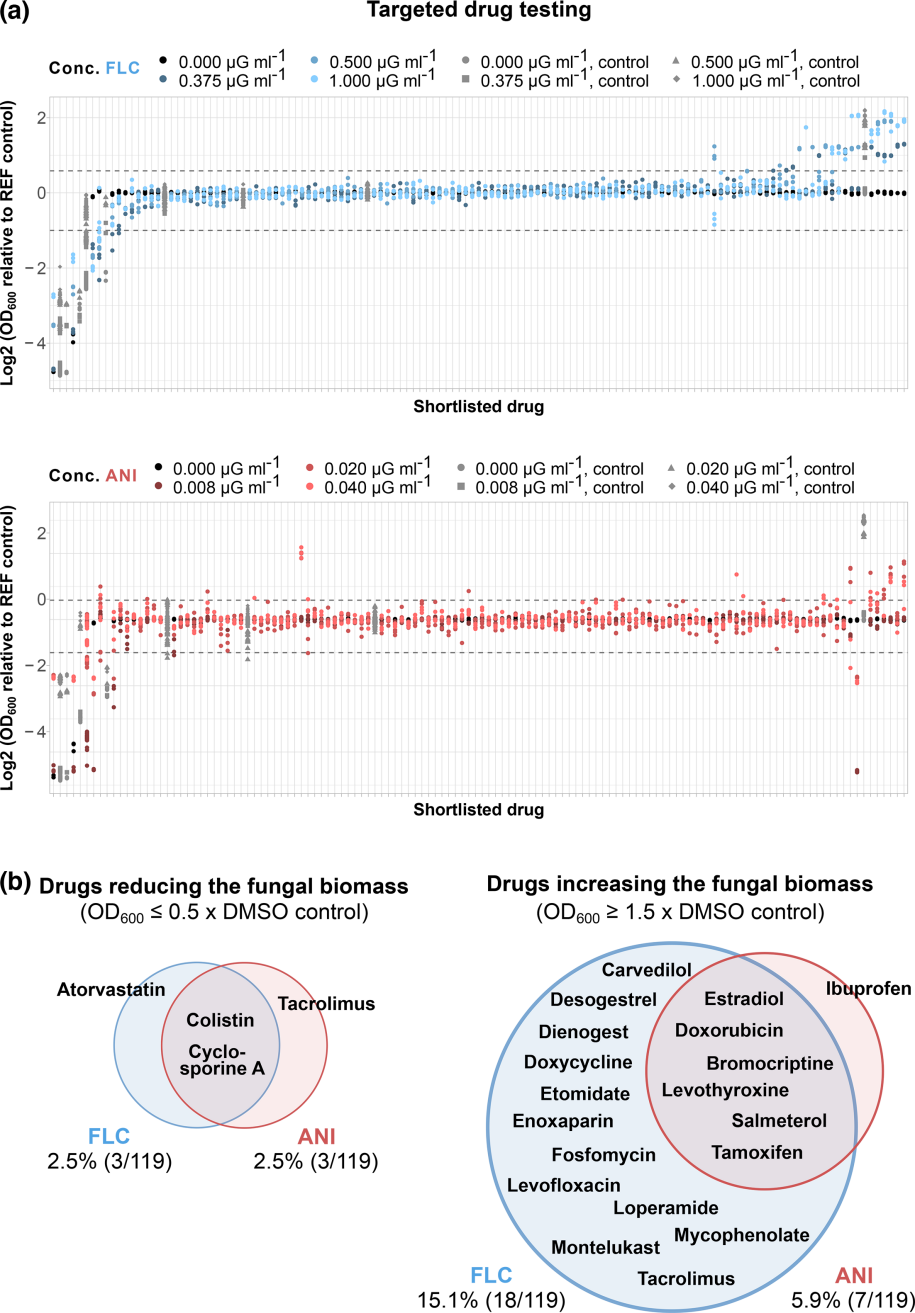
Targeted drug testing in *C. albicans* reveals negative and positive interactions of drugs directed against non-fungal diseases commonly administered in conditions with a risk of fungal infections. (**a**) Shortlisted drugs were exposed to *C. albicans* cultures, in the absence and presence of different concentrations of FLC (blue) and ANI (red). Fungal growth, quantified by measuring the OD_600_ after 60 h culturing, of the tested shortlisted drugs is displayed as Log2 of the relative growth compared with an reference (REF) control, being antifungal+DMSO of the matched condition. Grey dashed lines represent the threshold defining a ‘hit’. Ten micromolar of the antifungals amphotericin B, FLC and ANI were included in the tested compound shortlisted as a positive control for fungal growth suppression. X-axis labels are not displayed here due to space constraints but are provided in Fig. S2. Compounds were tested in *n*=3 technical replicates. (**b**) Hits identified from targeted drug testing. Interactions with FLC (blue) and ANI (red) classified as ‘hits’ altered the fungal OD by more than 0.5× and 1.5× mean of OD_600_ compared with the mean of the REF control (antifungal+DMSO).

Compounds were purchased from commercial suppliers (Table S2, available in the online Supplementary Material), diluted in DMSO to a working concentration of 1 mM and stored at −80 °C until usage. Compounds insoluble in DMSO were dissolved in sterile water on the day of usage to a concentration of 10 mM and diluted 1:10 in DMSO to a working concentration of 1 mM. The compound library was thawed on the day of the experiment, and controls were prepared, including SM only (untreated control), DMSO only (reference control, including antifungal+DMSO) and 90% DMSO/10% water (control water-soluble drugs). In addition, supra-MIC concentrations of FLC (sc-205698A, Santa Cruz Biotechnology), ANI (SML2288, Merck) and amphotericin B (sc-202462A, Santa Cruz Biotechnology) served as controls for fungal growth suppression and were used in final concentrations of 10 µM. To prepare 96-well edge plates, SM containing three concentrations of FLC (2×0.375, 2×0.5 and 2×1 µg ml^−1^), ANI (2×0.008, 2×0.02 and 2×0.04 µg ml^−1^) or an equivalent amount of DMSO (0 µg ml^−1^ FLC/ANI) was prepared and 98 µl of the antifungal- or DMSO-enriched medium was added to the plates. Subsequently, 2 µl of each compound from the library and corresponding controls were dispensed into the wells using the Biomek i5 robot (Beckman Coulter Life Sciences), resulting in a final concentration of 10 µM. Following a 4 h pre-culture in SM, *C. albicans* was diluted in SM to twice the inoculation concentration (diluted to OD_600_=0.005, roughly corresponding to a final number of 2×10^4^ cells in each well). Afterwards, 100 µl of the cell solution was added, followed by four times mixing in the 96-well plates. All drugs were cultured in triplicate. Additionally, untreated (no antifungal) and blank controls were incorporated in triplicate for each plate. The SM only, reference and DMSO/water controls were included as quintuplicate on every plate. All conditions underwent randomization through the Python shuffle() function. Edge effects were minimized by adding 1.5 ml sterile water to every edge in the 96-well edge plate. The plates were incubated at 30 °C and 60% humidity, and optical density (OD) at 600 nm was measured every 90 min for a duration of 72 h using a BioTek Epoch 2 microplate reader (Agilent Technologies) connected to a BioTek BioStack 3 microplate stacker (Agilent Technologies). The experimental set-up of targeted drug testing is displayed in (Fig. 1b) and (Fig. 2).

### Checkerboard assays

**Fig. 3.**
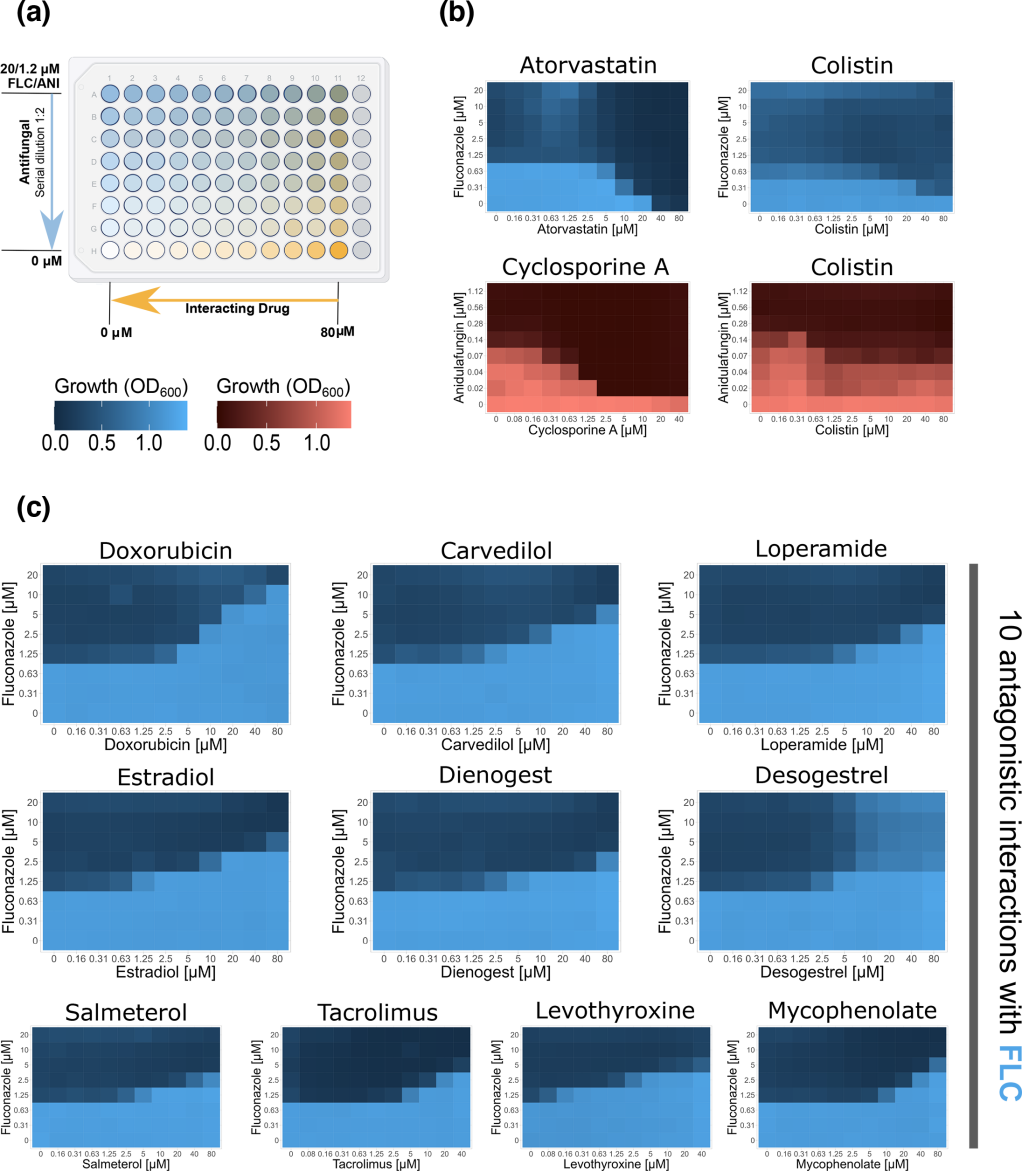
Checkerboard assays identify synergistic and antagonistic drug interactions with FLC and ANI. (**a**) Experimental set-up of checkerboard assays, including a concentration range of the antifungal (FLC or ANI) on the y-axis and concentration range of the interacting drug on the x-axis of a 96-well edge plate. (**b**) Checkerboards with FLC (blue) and ANI (red) showing synergistic interactions, identified by a reduction of at least two steps in the typical downward staircase pattern characteristic of synergy. (**c**) Checkerboards of ten drugs that increased the biomass of *C. albicans* show the typical staircase pattern of antagonistic DDIs, with at least two steps upwards. (**b**) and (**c**) display one of the experimentally conducted two biological replicates. All performed checkerboards are shown in Fig. S3. All checkerboards were tested with two biological replicates.

Drug interactions of the hits identified from targeted drug testing with FLC and ANI were evaluated by conducting checkerboard assays (Fig. 3a). A 10 mM stock of the hit compound was diluted in SM to 4×80 µM (excepting levothyroxine sodium, cyclosporine A and tacrolimus, which were diluted to 4×40 µM due to poor solubility). A 100 µl of the dissolved drug in SM was filled in a 96-well edge plate in column 11 and horizontally serial diluted (1:2) with SM, including all wells of columns 2–10. The remaining 50 µl of the drug was added to the blank positions (12A-H). Subsequently, antifungals were diluted in SM to four times the highest concentration of FLC or ANI, including 4×20 and 4×1.2 µM, respectively, and seven times serial diluted (1:2). Fifty microlitres of the dilutions were added to each well, while the highest concentrations of the antifungal were added to A1-12 and systematically lowered vertically. H1-12 were filled with SM only. After a 4 h pre-culture of *C. albicans*, cells were diluted in SM (OD_600_=0.005, roughly corresponding to 2×10^4^ cells/well), and 100 µl of the cell solution was added to well A-H1-11 in the 96-well edge plates. All blank positions (A-H12) were filled up with 100 µl SM. To minimize edge effects, all edges were filled with 1.5 ml sterile water. Plates were incubated at 30 °C and 60% humidity. OD_600_ was measured every 90 min for a duration of 72 h using a BioTek Epoch 2 microplate reader connected to the BioTek BioStak 3 microplate stacker. Two biological replicates of each checkerboard assay were prepared. Since most tested non-antifungals showed no intrinsic antifungal effect and could thus not be assigned to an MIC, synergism and antagonism were not assessed by calculating the fractional inhibitory concentration index (FICI), as is usually the case. Instead, the distinction of synergistic and antagonistic interactions from those that are additive was based on the examination of the characteristic staircase pattern of the interactions. Based on the Loewe additivity model, interactions were considered to be synergistic or antagonistic when both replicates of the assay exhibited at least two steps in the staircase pattern, roughly corresponding to an ≤0.5-fold (synergistic) or ≥4-fold (antagonistic) MIC that underlies the interpretation of the FICI [16].

### Disc diffusion assays

**Fig. 4.**
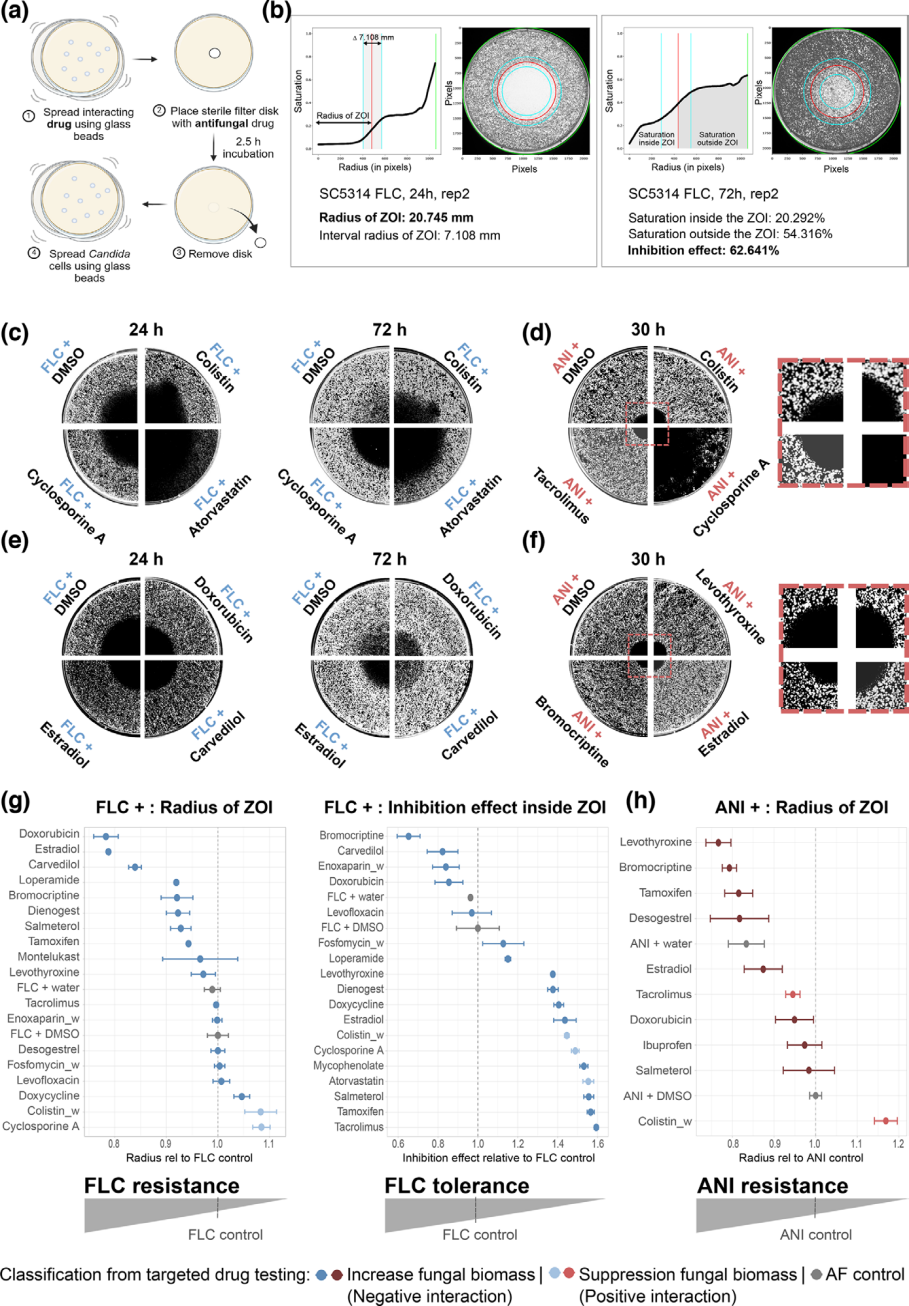
Drugs directed against non-fungal diseases increase antifungal resistance and tolerance in *C. albicans*. (**a**) To perform DDAs, the interacting drug was equally spread over a Petri dish, and, subsequently, a sterile filter disc containing the tested antifungal (FLC or ANI) was placed in the centre of the dish. After 2.5-h diffusion time, the disc was removed and fungal cells were equally spread over the dish. (**b**) Software output from evaluation of DDAs. Resistance was evaluated by calculating the inflection point of the saturation curve measured from the center of the ZOI outward at 24 h (FLC) or 30 h (ANI), reflecting the radius of the ZOI (red line). After 72 h, the inhibition effect, determined by the saturation inside the ZOI compared to the saturation outside the ZOI, served as an estimation for tolerance. The blue lines display the interval of the radius of the ZOI, while the green line highlights the edge of the petri dish. (**c**) Resistance (24 h) and tolerance (72 h) assessed by DDAs for positive interactions with FLC. (**d**) DDAs evaluating ANI resistance at 30 h for drugs that showed positive interactions with ANI during targeted drug testing. (**e**) DDAs for selected drugs that caused negative interactions with FLC during targeted drug testing, showing resistance at 24 h and tolerance at 72 h. (**f**) Representative negative interactions with ANI, evaluated for ANI resistance. (**c–f**) DDAs for all drugs tested, including all replicates (*n*=3 biological replicates), are shown in Fig. S5. (**g**) Many drugs alter resistance and tolerance to FLC (normalized to FLC+DMSO control). (**h**) Drugs that negatively interacted with ANI while targeted drug testing mainly also showed increased ANI resistance at 30 h in DDAs. (g, h) In two cases, resistance was strongly reduced (including DDAs for the interactions of atorvastatin+FLC and cyclosporine A+ANI) and caused such a dramatic loss of antifungal activity that no ZOI was detectable. Consequently, these data are not shown here. Similarly, desogestrel dramatically increased FLC tolerance, so that growth inside the ZOI could not be distinguished from the outer part of the ZOI; thus, these data are also not shown here (see Fig. S5). (g) and (h) with the drugs presented in a consistent order are shown in Fig. S6. *N*=3 biological replicates of all tested drug combinations, except for DMSO+FLC (*n*=6). ‘_w’ indicates that the drug was dissolved in water rather than DMSO.

To determine the effect of identified hits from targeted drug testing on antifungal resistance and tolerance, disc diffusion assays (DDAs) were performed (Fig. 4a) . The day before usage, 9 mm Petri dishes containing 14 ml SM agar were prepared. A 100 µl of the hit identified during targeted drug testing at 10 mM stock concentration was evenly spread over the dish utilizing ten glass beads (4 mm, 6×10 s shaking) and resulted in a final concentration of 70 µM after diffusing into the agar. Subsequently, an FLC or ANI disc containing 25 µG FLC or 5 µG ANI was placed in the centre of the Petri dish. To enable diffusion of the drugs into the agar, plates were incubated for 2–3 h at room temperature. After incubation, the disc was removed and 100 µl of the *C. albicans* culture (OD_600_=0.025, roughly corresponding to 1×10^5^ cells/Petri dish) was evenly distributed over the disc using ten glass beads (4 mm, 6×10 s shaking). Petri dishes were incubated for 72 h (30 °C, 60% humidity) and scanned after 24, 30, 48 and 72 h using an Epson Perfection V800 Photo scanner (SEIKO Epson CORPORATION). DDAs were performed as biological replicates, including a blank (no fungal cells), DMSO (DMSO on the disc instead of the antifungal) and reference (FLC/ANI on the disc+DMSO evenly spread on the plate instead of the hit) control. Antifungal resistance was automatically analysed by measuring the radius of the zone of inhibition (ZOI) at 24 h (FLC DDAs) and 30 h (ANI DDAs). Antifungal tolerance was computationally evaluated in scans after 72 h by quantifying the inhibition effect inside the ZOI and correlates with colony growth within the halo (Fig. 4b). To automate and streamline the estimation of the MIC in the DDAs, we developed a Python program that performs a radial analysis of growth patterns on the assay plate. The program applies concentric circular masks of a given thickness to summarize the gradient of growth density from the centre of the assay outward. The centre is determined by calculating the interpolated intersection of two perpendicular saturation curves formed by the sum over the X and Y voxel. This estimate is further refined using the signal from the diffusion disc, if present. Growth density within each circular mask is aggregated and smoothed using the Savitzky–Golay algorithm. The MIC radius is then estimated by identifying the peak of the first derivative of the resulting saturation curve, with the width of this peak corresponding to the intervals of the MIC estimation.

### *Galleria mellonella* survival infected with *C. albicans*

**Fig. 5.**
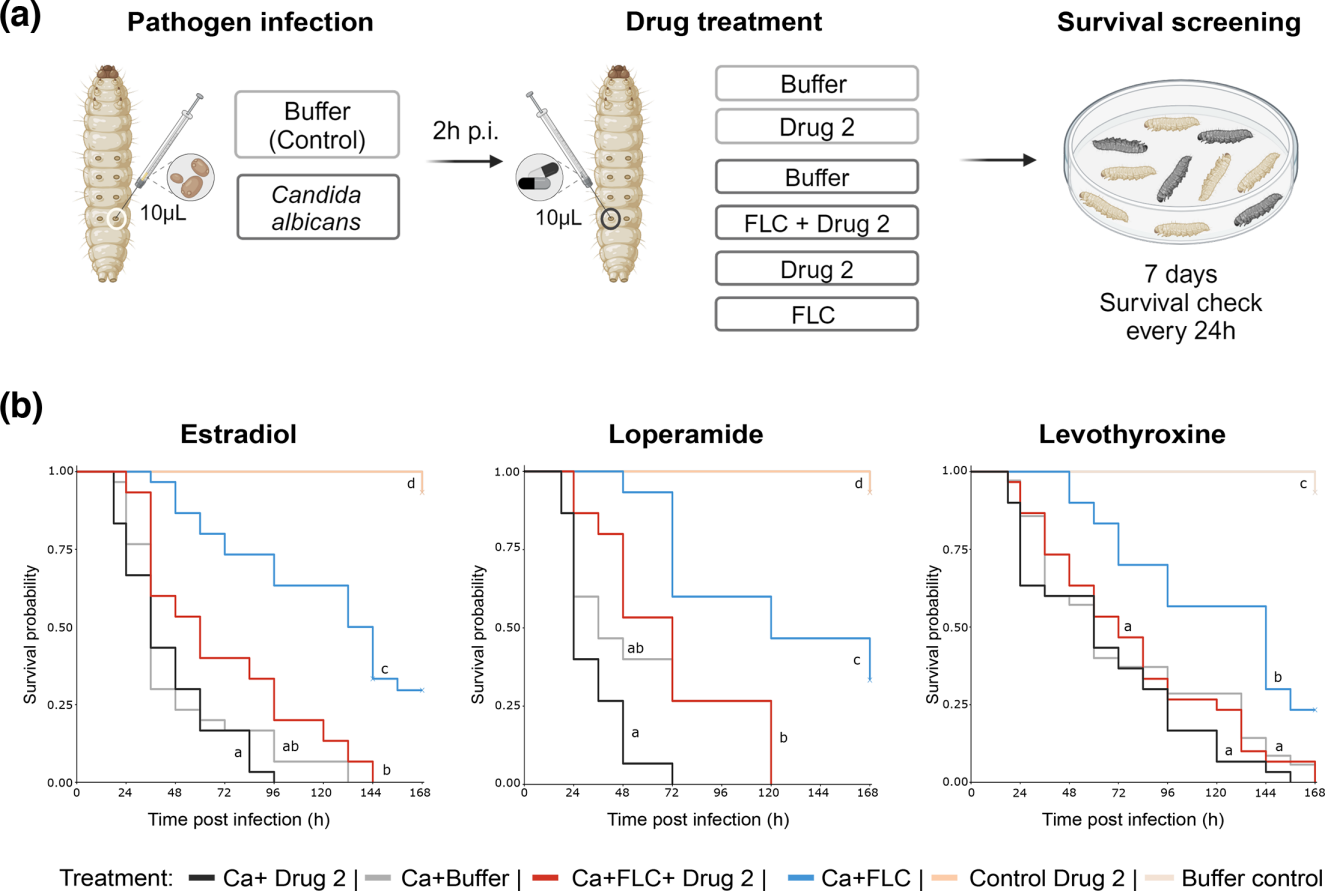
Impact of FLC antagonizers identified on *G. mellonella* survival during systemic fungal infection. (**a**) Experimental workflow of *G. mellonella* infection and drug treatment. Larvae of *G. mellonella* were injected with 5×10^5^ *C. albicans* cells. Two hours after infection, larvae were treated with either no drug (PBS/DMSO, Ca+Buffer, grey), FLC alone (Ca+FLC, blue), th antagonizing drug alone (Ca+Drug 2, black) or a combination of FLC+antagonizing drug (Ca+FLC+Drug 2, red). Additionally, control groups of larvae were injected with PBS/DMSO (Buffer control, light beige) and antagonizing drugs (Control Drug 2, dark beige) in the absence of fungal infection. After treatment, larvae were kept at 30 °C, and survival was monitored every 24 h. (**b**) Kaplan–Meier survival analysis of infected larvae showed significantly lower survival probabilities when infected larvae were treated with FLC and ethinyl estradiol, loperamide or levothyroxine (red) compared with FLC treatment alone (blue; Ca+FLC+estradiol vs Ca+FLC, *z*=4.770, *P*=2.76×10^−5^; Ca+FLC+levothyroxine vs Ca+FLC, *z*=3.854, *P*=0.001746; Ca+FLC+loperamide vs Ca+FLC, *z*=3.244, *P*=0.017681). Different treatments are represented by different colours. Survival was calculated as the living proportion of larvae from every treatment group. For each condition, *n*=15 replicates were included, while each experiment was conducted twice. Crosses indicate the presence of right-censored data (i.e. larvae that were not dead at the end of the experiment). Treatment marked with the same letter showed no significant difference when performing a Wilcoxon rank sum test followed by a Bonferroni correction to account for multiple comparisons. All statistical comparisons between each group are included in Table S3.

Antagonizing compounds with FLC identified from *in vitro* checkerboard assays were tested in an invertebrate model of *C. albicans* pathogenesis (Fig. 5a). For this purpose, *C. albicans* was directly injected into the haemolymph of *G. mellonella* larvae. This procedure results in a systemic infection that enables precise testing of antifungals and other clinically relevant drugs during infection [17,18]. Larvae were injected individually through the last right pro-leg into the haemocoel using a Hamilton Neurosyringe with 10 µl of *C*. *albicans* (5×10^7^ c.f.u. ml^−1^). Two hours post-infection (p.i.), larvae were treated with 10 µl of different drug combinations including FLC (4 mg kg^−1^)+antagonizing compound: carvedilol (sc-200157, Santa Cruz Biotechnology), desogestrel (Y0000509, Sigma), dienogest (Y0001785, Sigma), doxorubicin hydrochloride (sc-200923A, Santa Cruz Biotechnology), ethinyl estradiol (sc-205318, Santa Cruz Biotechnology), levothyroxine sodium (sc-235497, Santa Cruz Biotechnology), loperamide hydrochloride (sc-203116, Santa Cruz Biotechnology), mycophenolate mofetil (sc-200971A) or tacrolimus (SEL-S5003, Biozol). FLC (4 mg kg^−1^) and the antagonizing compound were dissolved in PBS with 10% DMSO. Due to the limited solubility of the antagonizing drugs in PBS/DMSO mixtures, the drugs were injected at saturation concentrations in 90% PBS/10% DMSO (injected drug concentration <1 mg ml^−1^). One milligramme of each compound was sonicated in 1 ml of PBS with 10% DMSO for 10 min. Following this, the drug-PBS/DMSO suspension was centrifuged at 14,000 r.p.m. for 10 min, and 900 µl of the supernatant was used for injection. Controls included: a drug toxicity control (uninfected larvae injected with the antagonizing compound alone), a buffer control (uninfected larvae injected with 90% PBS/10% DMSO), an untreated infection control (larvae infected with *C. albicans* and treated with 90% PBS/10% DMSO), an FLC-only control (larvae infected with *C. albicans* and treated with 4 mg kg^−1^ FLC) and a drug-only control (larvae infected with *C. albicans* and treated with the antagonizing drug alone without FLC). Fifteen larvae per group were used, and the experiment was repeated twice. After injection, larvae were placed in sterile 18 cm Petri dishes containing Whatman filter paper, incubated for 7 days at 30 °C and checked for survival every 24 h. Larvae that showed a brown-dark colour and no sign of movement when touched were considered to be dead. Mortality rates were calculated for each treatment. A Wilcoxon rank sum test was used to perform pairwise comparisons of mortality rate ranks between treatments and a Bonferroni correction to account for multiple comparisons.

## Results

### Mining health care guidelines to identify medications most commonly co-administered clinically during fungal infection comorbidities

We systematically mined the clinical guidelines collected by the AWMF in Germany (Arbeitsgemeinschaft der Wissenschaftlichen Medizinischen Fachgesellschaften, AWMF, https://www.awmf.org/ [14]) to identify pathologies or medical interventions that state a relationship with fungal infections (Fig. 1a). In a first step, the total set of 813 clinical guidelines accessible through the AWMF repository was obtained. These guidelines were parsed computationally for the presence of 75 terms related to fungal infections or their treatment, including ‘fungal infection’, ‘Candida’ or ‘antifungal’. At least one keyword was detected in 249 guidelines, amounting to approximately one-third of all screened guidelines.

In a second step, the 249 associations were manually assessed, for example, to exclude false positives. Guidelines were retained if they mentioned fungal infections in the context of other therapies, and if they addressed pathologies or medical interventions stated as increasing the risk of fungal infections, or if they could be caused by fungal infections (Table S1). The severity and type of fungal infection were not considered in the selection process, but guidelines were excluded if they had no medically relevant connection to fungal infections. In total, we obtained a set of 63 guidelines addressing the treatment of 40 pathologies or medical interventions where fungal infections often occur (Fig. 1a and Table S1). For example, the guideline on how to treat multiple myeloma noted invasive fungal infections as a severe complication and a prognostic factor for the severity of multiple myeloma and recommended an anti-infection prophylaxis dependent on the conducted therapy and individual risk factors (Table S1, AWMF guideline registration number 018/0350L [19]).

Next, we used the selected guidelines to extract the list of (non-antifungal) medications used as part of the management in these pathologies. We ranked the compounds based on the frequency of occurrence and also prioritized different drug classes to cover multiple mechanisms of action. This process resulted in a shortlist of 119 compounds (Table S2), which, for instance, are used in oncology and transplantation medicine, but also drugs that are less commonly associated with a fungal health burden disease, such as muscle relaxants or antihistamines. Furthermore, a significant proportion of the drugs reflect the common problem of fungal–bacterial and fungal–viral co-infections (Fig. 1a, ‘Infectious diseases’), and thus, the list also included antibacterials and antivirals (*n*=35, Fig. 1a, ‘Anti-infectives’).

### Testing the effect of clinically used compounds on interactions with antifungals

The 119 tested compounds were obtained commercially (Table S2) and dissolved in DMSO or water, depending on their solubility properties. Then, we measured their effect on the growth of *C. albicans*’ reference strain SC5314 compared with a vehicle control, the compound administered alone (compound control) and in combination with the antifungals FLC and ANI. This setup thus allowed us to test for potential DDIs with an azole and an echinocandin drug, respectively, which are often the first-line drug classes used to treat systemic *C. albicans* infections [20]. Antifungal concentrations used ranged from no drug to below and above the MIC50 (Fig. 1b), which was experimentally determined in broth dilution assays before. Fungal growth (OD_600_) was measured over 60 h culturing, and drug combinations of compound and antifungal were classified as a ‘hit’ if the mean of all tested replicates (*n*=3) enhanced or reduced the biomass of the fungal cultures by 1.5- or 0.5-fold compared with the mean of the control (antifungal+vehicle) in the same growth condition.

#### Effect of targeted clinical compounds: intrinsic antifungal activities and synergism with FLC and ANI

Two of the 119 tested compounds, sirolimus and octenidine dihydrochloride, had intrinsic antifungal activity and reduced *C. albicans* growth without an added antifungal drug (Fig. S1). Further, 22 compounds (~18% of all tested compounds) altered *C. albicans* growth by enhancing or reducing the fungal biomass when given in combination with an antifungal drug (Figs 2a, b and S2). Of these, *n*=12 (~55%) affected *C. albicans* only in combination with FLC, one compound, being ibuprofen, was effective only in the presence of ANI and *n*=9 (~41%) affected growth in combination with both, FLC and with ANI (Fig. 2b).

Four compounds (~3% of all tested compounds) increased the antifungal activity of FLC and/or ANI. These drugs included two immune-suppressing calcineurin inhibitors (cyclosporine A and tacrolimus [21]), one HMG-CoA reductase inhibitor (atorvastatin [22]) and one antimicrobial that targets membrane integrity in Gram-negative bacteria (colistin sulphate [23], Fig. 2b). Checkerboard assays of these positive interactions (Fig. 3a) showed that, as seen in prior studies [24,26], atorvastatin and colistin sulphate interacted synergistically with FLC and that cyclosporine A and tacrolimus interacted synergistically with ANI (Fig. 3b). The other drug-antifungal combinations, including cyclosporine A-FLC and colistin sulphate-ANI, did not show definitive synergistic interactions (Fig. S3a).

#### Ten drugs directed against non-fungal diseases antagonize FLC

Notably, *n*=19 (~16%) of the tested compounds decreased the efficacy of the antifungals. Of these, *n*=12 compounds increased *C. albicans* growth at antifungal concentrations above the MIC of only FLC, *n*=1 of only ANI and *n*=6 of both FLC and ANI (Fig. 2b). These negative interactions included several drug classes and different mechanisms of action. For example, tamoxifen and tacrolimus fully restored fungal growth at every FLC concentration tested (Fig. S4). In combination with ANI, ibuprofen caused the highest restoration of fungal growth, with up to ~70% quantified biomass of the untreated control (Fig. S4). Amongst the hits were modulators of steroid hormone synthesis, including the synthetic progesterones dienogest and desogestrel [27], ethinyl estradiol (a *β*-estradiol derivative [28]) and tamoxifen (a selective oestrogen receptor modulator [29]). Two additional compounds disrupt DNA and RNA synthesis in prokaryotic (the antibacterial levofloxacin [30]) or eukaryotic (the antineoplastic doxorubicin [31]) cells. Two other identified drugs are prominent *β*-receptor modulators: the non-selective *β*-blocker carvedilol [32] and the *β*_2_-receptor agonist salmeterol [33].

As for the positive interactions, we conducted checkerboard assays to distinguish antagonistic and additive interactions. Drug interactions that produced a characteristic staircase pattern and resulted in a ≥ ~4-fold change in MIC were defined as antagonistic. Based on these criteria, ten compounds were identified as having antagonistic interactions with FLC (Fig. 3c, Table 1). These included the hormonal contraceptives ethinyl estradiol, desogestrel and dienogest as well as mycophenolate mofetil and tacrolimus (immune suppressors), carvedilol (an antihypertensive), salmeterol xinafoate (a bronchospasmolytic), doxorubicin hydrochloride (an antineoplastic), loperamide hydrochloride (an antidiarrheal) and levothyroxine sodium (a synthetic thyroid hormone). None of the compounds passed the ≥4-fold MIC change threshold with ANI (Fig. S3b).

**Table 1.** Compounds antagonistic to FLC, used against non-fungal diseases, affecting the antifungal responses of *C. albicans*

Compound name	Suggested MOA	Relevant health condition	FLC^res^ (24 h)	FLC^tol^ (72 h)	Increase *G. mellonella* mortality	Relevant citation
**Ethinyl estradiol**	*β*-Estradiol derivate	Contraceptive	**↑**	**↓**	**Yes** (*P*<0.001)	Steroid hormones oestrogen and progesterone protect *Candida* cells from FLC by increased *CDR1* gene expression [56,57]
**Desogestrel**	Synthetic gestagen	Contraceptive	**→**	**↑**	No	
**Dienogest**	Synthetic gestagen	Contraceptive	**↑**	**↓**	Not statistically significant	
**Levothyroxine sodium**	Synthetic thyroxine (T4)	Treatment hypothyroidism	**→**	**↓**	**Yes** (*P*<0.01)	Previous drug screen also reports reduced antifungal effect of FLC on fungal growth [12]
**Loperamide hydrochloride**	Opiate receptor modulator	Chemotherapy-related diarrhoea	**↑**	**↓**	**Yes** (*P*<0.01)	None
**Doxorubicin hydrochloride**	Topoisomerase II inhibitor	Antineoplastic agent	**↑**	**↑**	No	Increased CDR1 and CDR2 gene as well as Cdr1p and Cdr2p protein expression [58]
**Salmeterol xinafoate**	Adrenergic *β*_2_-receptor agonist	COPD and asthma	**↑**	**↓**	No	Previous drug screen also reports reduced antifungal effect of FLC on fungal growth [12]
**Carvedilol**	Non-selective *β*-blocker	Liver cirrhosis and rosacea	**↑**	**↑**	Not statistically significant	Previous drug screen reports reduced fungal growth after 24 h [12]
**Mycophenolate mofetil**	Inhibitor Inosine-50-monophosphate dehydrogenase	Transplantation (immune suppression)	na	**↓**	No	Antagonism of MPA+FLC in *C. glabrata* [59]
**Tacrolimus**	Calcineurin inhibitor	Transplantation (immune suppression)	**↑**	**↓**	Not statistically significant	Clears tolerance *in vitro*, presumably via stress responses [34,36]

FLCres and FLCtol: referring to the change in FLC resistance and FLC tolerance, respectively, compared with the reference control.

COPD, Chronic obstructive pulmonary disease; MOA, mechanism of action; MPA, mycophenolic acid; na, not sufficient cell growth after 24 h to measure the RAD.

### Drugs directed against non-fungal diseases affect both antifungal resistance and tolerance

In our study, we followed the definition of fungal drug resistance and tolerance as introduced by Rosenberg *et al*. [34]. Therein, drug resistance is defined as an increase in the MIC and tolerance as the slow growth of subpopulations at any drug concentration above the MIC. In DDAs, this definition corresponds to a decrease in the ZOI radius (RAD) in case of resistance and to an increase in growth inside the ZOI, also known as the fraction of growth (FoG), in case of tolerance. This definition of resistance and tolerance captures both genetic and conditional causes of altered drug resilience, such as DDIs. To distinguish whether the DDIs we detected affected FLC resistance and/or tolerance, we performed DDAs (Fig. 4a, b).

The three drugs exhibiting positive interactions with FLC (atorvastatin, colistin sulphate and cyclosporine A) resulted in increased RAD levels relative to FLC alone, and FoG levels were cleared to background levels (Figs 4c, g, S5 and S6). Thus, these compounds reduced FLC resistance and tolerance levels. For drugs that positively interacted with ANI, two of the three compounds (colistin sulphate and cyclosporine A) increased RAD, indicating a reduced resistance, while tacrolimus slightly reduced RAD, indicating some increase in resistance (Figs 4d, h, S5 and S6). Since the reference strain SC5314 exhibits little to no intrinsic tolerance against ANI, the potential impact of the tested drugs on ANI tolerance was not assessed.

Further, some drugs that had negative interactions with antifungals were accompanied by a reduced RAD compared with the antifungal only, indicating increased resistance. In particular, bromocriptine mesylate, carvedilol, dienogest, doxorubicin hydrochloride, ethinyl estradiol, loperamide hydrochloride, montelukast sodium, salmeterol xinafoate and tamoxifen increased FLC resistance (Figs 4e, g, S5 and S6), while bromocriptine mesylate, doxorubicin hydrochloride, ethinyl estradiol, levothyroxine sodium and tamoxifen increased ANI resistance (Figs 4f, h, S5 and S6). Drugs that did not show a reduced RAD, thus not indicating increased antifungal resistance, were enoxaparin sodium, doxycycline hyclate, desogestrel, fosfomycin disodium, levofloxacin hemihydrate, levothyroxine sodium and tacrolimus in combination with FLC (Fig. 4g), as well as ibuprofen and salmeterol xinafoate combined with ANI (Fig. 4h). Interestingly, FLC tolerance assessed from drugs co-administered that caused negative DDIs with FLC showed different effects. Most drugs – including dienogest, doxycycline hyclate, ethinyl estradiol, levofloxacin hemihydrate, levothyroxine sodium, mycophenolate mofetil, salmeterol xinafoate, tacrolimus and tamoxifen – reduced or even completely removed FLC tolerance by suppressing subpopulation growth in the ZOI. Conversely, five drugs – including bromocriptine mesylate, carvedilol, desogestrel, doxorubicin hydrochloride and enoxaparin sodium – increased the FoG inside the ZOI of FLC, thus increasing FLC tolerance (Figs 4e, g, S5 and S6). Overall, negative DDIs that antagonized FLC generally also increased FLC resistance (except desogestrel), while FLC tolerance was either increased or reduced, depending on the compound.

### FLC antagonists on *C. albicans*-infected *G. mellonella* larvae

Next, we tested the impact of drugs identified as antagonistic to FLC on *C. albicans* in *G. mellonella*, chosen as a non-mammalian *in vivo* model to test whether the interaction between a compound directed against non-fungal diseases and the fungal pathogen has the potential to affect antifungal therapy in an *in vivo* setting. *G. mellonella* larvae were injected with *C. albicans* and subsequently treated with FLC alone or in combination with a FLC antagonist (injected drug concentration <1 mg ml^−1^), i.e. carvedilol, dienogest, desogestrel, doxorubicin hydrochloride, ethinyl estradiol, levothyroxine sodium, loperamide hydrochloride, mycophenolate mofetil, salmeterol xinafoate or tacrolimus, respectively (Fig. 5a). With the exception of desogestrel and mycophenolate mofetil, these drugs all limited the efficacy of the azole antifungal and resulted in a decreased median survival time (time point when no signs of viability in 50% of treated larvae) when combined with FLC, compared with FLC alone (Fig. S7a). Moreover, co-administration of the drugs ethinyl estradiol, levothyroxine sodium and loperamide hydrochloride with FLC resulted in significantly higher mortality compared with those treated with FLC alone (Fig. 5b). Neither the solvent control (PBS/DMSO) nor administration of the antagonizing compound alone (Control Drug 2) affected larval survival in the absence of a fungal infection. For carvedilol, salmeterol and tacrolimus, our data revealed no statistically significant changes in survival in our experiment, but the trajectories were not identical, suggesting that drug interactions in other settings, i.e. *in vivo*, should not be ruled out and that future studies might be warranted (Fig. S7b). The addition of mycophenolate mofetil, doxorubicin hydrochloride and dienogest to FLC did not impair *G. mellonella* survival at the concentrations tested. Notably, desogestrel, which antagonized FLC *in vitro*, slightly improved *G. mellonella* survival when combined with FLC relative to the FLC treatment alone (Fig. S7b). Thus, co-administration to FLC of three out of ten tested drugs which were antagonistic with FLC *in vitro* significantly increased larval mortality in a non-mammalian *in vivo* model for invasive candidiasis. A summarizing overview of all antagonizing drugs including their effect on FLC treatment in infected *G. mellonella* is shown in Table 1.

## Discussion

While many fungal infections remain mild, the risk of invasive bloodstream infections is elevated in specific patients, for instance, those with comorbidities, a weakened immune system, surgery or severe wounding [3,6, 7]. In these cases, treatment failures and mortality can be high. For example, mortality is ranging from 30 to 80% in *Candida* bloodstream infections [6,7, 35]. Despite these high therapy failure rates, drug resistance, a common cause of treatment failure in bacteria, is only observed in a small number (<1%) of *C. albicans* infections [36] but is considered to be on the rise. Meanwhile, antifungal drug tolerance, in which subpopulations can grow slowly in the presence of supra-MIC antifungal drug concentrations, is much more common. Tolerance is particularly frequently observed against azole antifungals [34] at body temperature [37] and may underpin antifungal treatment failure [5,36]. Thus far, we understand little about how antifungal drug resistance and tolerance evolve during severe fungal infections and which factors influence this property.

Because fungal infections often become problematic upon medical procedures or as comorbidities in patients with poor immune status [6], antifungal therapies are rarely supplied alone to these patients, but rather frequently co-administered on top of the primary therapies. In this study, we examined whether the co-administration of medications used in disease with a risk of fungal infections could affect fungal drug responses in the pathogen. Indeed, fungal and mammalian cells share many biochemical pathways [11,38], which create a potential that a drug directed against a human target also has an effect on the fungus. We focused on identifying drugs with ‘real-world’ relevance, by performing a systematic search of current clinical guidelines that reference a fungal infection or an antifungal therapy. Since these guidelines are not written in a common or systematic structure, we chose a two-step approach. First, we computationally parsed the guidelines for the presence of antifungal therapy-related terms, and then, we manually curated the identified guidelines for those where fungal infections indeed play a significant role. While we cannot rule out false negatives, i.e. we miss those pathologies for which the guideline does not mention fungal comorbidity, or those for which no guideline document is available, this approach offered the possibility for drug prioritization amongst medications commonly used in routine clinical setting and in the context of fungal infections. As a result, our research mirrors the co-administration of medications to large numbers of patients across various medical fields. Notably, this systematic strategy reflected the scale of fungal infection comorbidities; 63 guidelines of various diseases contained a reference to an increased risk of fungal infection or recommended antifungal prevention. Moreover, with this focus on negative or antagonizing interactions, our strategy was complementary to drug repurposing approaches, in some of which FDA-approved compound libraries (e.g. the Prestwick Chemical Library) were screened for synergism with antifungals on *C. albicans*. Of note, we define antifungal resistance relative to the control (antifungal+DMSO), rather than based on clinical breakpoints (e.g. from standardized CSLI or EUCAST guidelines [39,40]), measured in different assays. Our study might thus detect interactions that are not seen in the standard assays applied in the clinic.

Testing 119 compounds selected in this strategy, we report an extremely high hit rate, where ~15% and ~6% of the tested compounds co-administered with FLC or ANI, respectively, affected – mostly negatively – the *C. albicans* antifungal response. Overall, FLC was more prone to be influenced by other drug treatments. This observation is consistent with previous studies that find considerably less DDIs with caspofungin compared with FLC [11]. Remarkably, FLC is approved for treating several *Candida* infections, including vaginal and systemic infections, and is crucial for prophylaxis of candidiasis [41]. A deeper understanding of the nature of these drug responses could thus be beneficial for optimizing treatment outcomes with azole antifungals.

Notably, a small number of drugs synergized with the antifungals and reduced antifungal resistance or exhibited antifungal properties by themselves. This included compounds like sirolimus and octenidine for which antifungal activity is well documented [25,34, 42,44], giving confidence in our approach. Indeed, sirolimus was initially discovered in a screen for antimicrobial properties measured by the diameter of the ZOI in solid agar assays [45,46]. However, although extensively discussed in literature [47], sirolimus has never been clinically introduced as an antifungal agent due to its immunosuppressive effects and is currently used primarily as an immunosuppressant [48]. Another example is colistin, which displayed synergistic interactions with FLC and ANI [25,49]. The drug was previously shown to increase the membrane permeability of FLC-treated *C. albicans* by binding to membrane lipids. These lipids are present in the membrane of ergosterol-depleted (FLC-treated) cells [25].

For ten of the drugs, we report antagonistic interactions with FLC, which manifested in at least two drug interaction assays. To the best of our knowledge, our study is the first report of antagonistic effects of FLC with mycophenolate, tacrolimus, carvedilol and loperamide in *C. albicans*. Doxorubicin, salmeterol and levothyroxine were previously reported to counteract FLC [10,12].

Our shortlist included the immunosuppressants mycophenolate and tacrolimus because of their role in maintenance therapy after organ transplantation. Both drugs also weaken endogenous immune defences against infectious diseases [50,51]. Indeed, antifungal stewardship guidelines recommend co-administration of FLC as a prophylaxis against fungal infections in certain circumstances following solid organ transplantation [52]. Our data revealed that both drugs reduced FLC susceptibility. Notably, tacrolimus has previously been described to reduce the MIC of *C. albicans* against FLC. This effect was strain-specific, with the most potent effects reported in FLC-resistant strains [53], which may account for the discrepancy with our findings. We thus suggest future studies about the use of FLC in patients treated with mycophenolate and tacrolimus.

Loperamide, identified here as an FLC antagonist, is an opioid receptor agonist. It is frequently used to treat chemotherapy-related diarrhoea [54], one of the most prevalent adverse events occurring in up to 80% of patients undergoing chemo- or radiotherapy [55]. Notably, this first-line treatment for chemotherapy-related diarrhoea is freely available over the counter in many countries and is usually consumed in extremely high doses (up to 12 and 16 mg per day for prophylactic and acute treatment, respectively) [54]. Also in this case, our study prompts for future investigations into the use of FLC in patients receiving loperamide therapy.

Another candidate highlighted by our investigations is desogestrel, a progestogenic steroid, that was the only FLC antagonist that promoted FLC tolerance rather than resistance. Here, we did not observe significant changes to viability when desogestrel was administered with FLC to *G. mellonella* larvae. However, the strong *ex vivo* effect allows speculating that the DDI could potentially influence drug tolerance and resistance in other conditions.

Drug-induced changes in resistance and tolerance might pave the way for the evolution of intrinsically resistant or tolerant strains. Indeed, while intrinsic antifungal resistance is relatively rare in *C. albicans* strains, several of the drugs tested here promoted the acquisition of increased levels of FLC resistance as well as a few examples of increased ANI resistance. The mechanism by which these drugs affect the antifungal response in *C. albicans* should be further investigated. We speculate that a large number of the interacting compounds might influence the cellular metabolism and thereby alter cells’ general growth and resilience, that unspecific drug–protein interactions might stimulate efflux and thereby reduce the intracellular drug concentrations of FLC, similar to what has been previously shown for doxorubicin [52], or that a number of compounds might influence the cell membrane or cell wall composition. Further, while the role of antagonistic interactions in the evolution of drug resistance or tolerance needs further study, it is important to highlight that the drugs tested herein are co-administered with antifungals in millions of individuals. One concern is that many of the FLC antagonists we report – including ethinyl estradiol, desogestrel, dienogest, salmeterol, carvedilol, mycophenolate and tacrolimus – are medications used in long-term disease management. It is worth speculating that prolonged usage of these antagonizing drug combinations might create conditions that increase fungal cell numbers during antifungal therapy and thereby favour the evolution of new traits within opportunistic fungal populations. Those new traits that provide a selective advantage could then tip the scales and promote drug resistance and/or tolerance in fungal pathogens within vulnerable patients.

In summary, our study reveals a high frequency of interactions between commonly administered medications amongst patients prone to fungal infections and the prevalent fungal pathogen *C. albicans*. Notably, many of the negative or antagonistic interactions reported involve medications that are standard treatments for conditions with a high risk of fungal infections and are frequently used in the long-term management of chronic diseases. The prevalence of these interactions suggests that polypharmacy may be an underestimated factor in the evolution of antimicrobial tolerance or resistance in fungal pathogens. Furthermore, our findings underscore the need for additional studies to evaluate the use of FLC in patients receiving mycophenolate, tacrolimus or loperamide and to determine whether these drug combinations compromise therapeutic outcomes.

## Supplementary material

10.1099/jmm.0.002046 Uncited Supplementary Material 1.

## Abbreviations

ANI: anidulafungin
AWMF: Association of the Scientific Medical Societies
DDAs: disc diffusion assays
DDIs: drug–drug interactions
FLC: fluconazole
FoG: fraction of growth
ON: overnight
SM: synthetic minimal medium
ZOI: zone of inhibition
