# Supplementary material for: Non-antifungal medications administered during fungal infections drive drug tolerance and resistance in Candida albicans
Source: J Med Microbiol. 2025 Jul 28;74(7):002046. doi: 10.1099/jmm.0.002046 (PMC12451754; doi:10.1099/jmm.0.002046)

1. Supplementary Tables

Supplementary Table 1:

Guidelines selected as “hits” after systematic keyword search in AWMF guidelines.

AWMF guideline registration number	Guideline published/ updated	Guideline topic
**Guidelines including pathologies and medical interventions which indicated an association with fungal infections**		
006-052	2021	Phimosis in children and adolescents
007-006	2016	Odontogenic infections
007-096	2017	Dental restoration and hear valve replacement
011-021	2020	Extracorporeal circulation (ECLS/ ECMO)
011-022	2019	Mediastinitis
013-001	2021	Psoriasis vulgaris
013/007	2019	Anal eczema
013-012	2012	Hidradenitis suppurative / acne inversa
013-028	2022	Urticaria
013-033	2019	Tinea Capitis
013-065	2022	Rosacea
013-048	2017	Dermatoses associated with dermal lymphostasis
013-091	2020	Pyoderma Gangrenosum
018/0350L	2022	Monoclonal gammopathy, multiple myeloma
018-037	2022	COVID-19 in oncology and haematology
020-018	2017	Cystic fibrosis
020-019	2022	Tuberculosis
021-017	2018	Liver cirrhosis
021-027	2017	Autoimmune liver disease
023-046	2019	Heart transplantation
026-022	2013	Lung disease in cystic fibrosis patients
030-044	2021	HIV-1-associated neurological disorders
030-050	2021	MOG-IgG associated disorders
030-098	2018	Cerebral Thrombosis
042-004	2016	Visceral leishmaniasis
048-014	2016	Oncology
057-017	2018	Diabetes mellitus
073-004	2018	Nutrition on ICU
079-001	2018	Sepsis
082-006	2017	Parental antibacterials
088-001	2018	Surgery obesity and metabolic diseases
112-001	2017	Primary immunodeficiency
113-001	2022	COVID-19
151-001	2020	Spondylodiscitis
166-004	2021	Urinary tract infection in paediatrics

189-001	2019	Primary antibody deficiency
018-015	2019	Hormonal contraception
032-054	2020	Supportive treatment in oncology
**Guidelines including pathologies caused by fungi**		
013-048	2022	Chronic Pruritus
013-063	2020	Pruritus ani
015-071	2013	Mastitis in breastfeeding population
017-024	2015	Tonsillitis
017-049	2019	Rhinosinusitis
017-066	2019	Antibacterial therapy in head and throat infections
020-003	2019	Cough
020-009	2017	Asthma
020-013	2017	Nosocomial pneumonia
020-020	2021	Pneumonia
021-024	2015	Infectious gastritis
022-004	2015	Infections of the central nervous system
023-024	2014	Infectious endocarditis
024-008	2021	Bacterial infections
024-025	2015	Neonatal sepsis
027-070	2021	Back pain in children and adolescents
030-108	2021	Brain
030-0450OL	2017	Breast cancer
043-044	2017	Urinary tract infection
048-011	2019	HIV therapy in children and adolescents
083-001	2018	Urinary tract
053-009	2014	Earache
053-010	2020	Sore Throat
Nvl-002	2020	Asthma
082-005	2020	*Candida* infections

Supplementary Table 2:

Shortlisted drugs purchased from commercial suppliers.

	Compound	Company	Product Number	Solvent
1	**Abacavir sulphate**	TargetMol	T6367	DMSO
2	**Acarbose**	Santa Cruz Biotechnology	sc-203492	DMSO
3	**Acetaminophen**	Sigma	PHR1005	DMSO
4	**Acetylsalicylic acid**	Sigma	A5376	DMSO
5	**Acyclovir**	Santa Cruz Biotechnology	sc-202906	DMSO
6	**Ambroxol hydrochloride**	Santa Cruz Biotechnology	sc-200816	DMSO
7	**Amethopterin**	Santa Cruz Biotechnology	sc-214534	DMSO
8	**Amlodipine**	Santa Cruz Biotechnology	sc-200195	DMSO
9	**Amoxicillin trihydrate**	Santa Cruz Biotechnology	sc-353009	DMSO
11	**Ampicillin**	Santa Cruz Biotechnology	sc-210812	DMSO
14	**Atorvastatin**	Sigma	PHR1422-1G	DMSO
15	**Atenolol**	Sigma	A7655-1G	DMSO
16	**Azathioprine**	Santa Cruz Biotechnology	sc-210853	DMSO
17	**Azithromycin**	TargetMol	TGM-T6401-50MG	DMSO
18	**Baricitinib**	TargetMol	TGM-T2485-25MG	DMSO
19	**Bictegravir**	TargetMol	T4493-10mg-TM	DMSO
20	**Bromocriptine mesylate**	Santa Cruz Biotechnology	sc-200395	DMSO
22	**Canagliflozin**	TargetMol	TGM-T1782-50MG	DMSO
23	**Capecitabine**	Santa Cruz Biotechnology	sc-205618	DMSO
24	**Carvedilol**	Santa Cruz Biotechnology	sc-200157	DMSO
25	**Ceftriaxone disodium salt**	Santa Cruz Bitoechnology	sc-211050	DMSO
26	**Cefuroxime sodium**	TargetMol	TGM-T1224-100MG	DMSO
27	**Cetirizine dihydrochloride**	Sigma	89126-50MG-F	DMSO
29	**Ciprofloxacin HCl**	Santa Cruz Biotechnology	sc-29064	DMSO
30	**Cisatracurium besilate**	Sigma	Y0001766	DMSO
31	**Clindamycin hydrochloride**	TargetMol	TGM-T6448-50MG	DMSO
32	**Clopidogrel sulphate**	Santa Cruz Biotechnology	sc-337638A	DMSO
33	**Cyclophosphamide monohydrate**	Sigma	C0768	DMSO

34	**Cyclosporin A**	Santa Cruz Biotechnology	sc-3503	DMSO
35	**Dapagliflozin**	TargetMol	T2389	DMSO
36	**Desogestrel**	Sigma	Y0000509	DMSO
37	**Dexamethasone**	Santa Cruz Biotechnology	sc-29059	DMSO
38	**Diclofenac sodium**	Santa Cruz Biothechnology	sc-202136	DMSO
39	**Dienogest**	Sigma	Y0001785	DMSO
40	**Dobutamine hydrochloride**	Sigma	D2954000	DMSO
41	**Docetaxel**	Biomol	CDX-D0371-M025	DMSO
42	**Dolutegravir sodium**	TargetMol	TGM-T2329-25MG	DMSO
43	**Domperidone**	Santa Cruz Biotechnology	sc-203032	DMSO
44	**Doxorubicin hydrochloride**	Santa Cruz Biotechnology	sc-200923A	DMSO
45	**Doxycycline hyclate**	Santa Cruz Biotechnology	sc-204734B	DMSO
46	**Empagliflozin**	Santa Cruz Biotechnology	sc-482194	DMSO
47	**Emtricitabine**	Santa Cruz Biotechnology	sc-207617	DMSO
48	**Epinephrine**	Sigma	E4250	DMSO
49	**Ethinyl estradiol**	Santa Cruz Biotechnology	sc-205318	DMSO
50	**Etomidate**	TargetMol	TGM-T1089-25MG	DMSO
52	**5 - Fluorouracil**	Santa Cruz Biotechnology	sc-29060	DMSO
55	**Furosemide**	Santa Cruz Biotechnology	sc-203961	DMSO
56	**Ganciclovir**	Thermo Fisher Scientific	461710010	DMSO
57	**Glyburide**	Santa Cruz Biotechnology	sc-200982	DMSO
58	**Hydrocortisone**	Santa Cruz Biotechnology	sc-300810	DMSO
59	**Ibuprofen**	Sigma	PHR1004	DMSO
60	**Imatinib mesylate**	Santa Cruz Biotechnology	sc-202180	DMSO
61	**Isoniazid**	TargetMol	TGM-T0972-50MG	DMSO
62	**Isoflurane**	Santa Cruz Biotechnology	sc-470926	DMSO
64	**Ketamine hydrochloride**	Sigma	K2753	DMSO
65	**Lamivudine**	Santa Cruz Biotechnology	sc-221830A	DMSO
66	**Levofloxacin hemihydrate**	Santa Cruz Biotechnology	sc-211735	DMSO
67	**Levothyroxine sodium**	Santa Cruz Biotechnology	sc-235497	DMSO
68	**Lisinopril**	Santa Cruz Biotechnology	sc-205378	DMSO
69	**Loperamide hydrochloride**	Santa Cruz Biotechnology	sc-203116	DMSO

70	**Losartan carboxylic acid**	TargetMol	T3461-25mg-TM	DMSO
71	**Melphalan**	Santa Cruz Biotechnology	sc-204799	DMSO
72	**Metamizole sodium**	Sigma	PHR1800	DMSO
73	**Metoclopramid**	Sigma	32473	DMSO
74	**Metformin hydrochloride**	Sigma	PHR1084	DMSO
75	**Metoprolol succinate**	Target Mol	TGM-T3165-50MG	DMSO
76	**Molnupiravir**	Biozol	CMS-CS-0114880	DMSO
77	**Montelukast sodium**	Santa Cruz Biotechnology	sc-202231A	DMSO
78	**Moxifloxacin hydrochloride**	TargetMol	TGM-T0331-50MG	DMSO
79	**Mycophenolate mofetil**	Santa Cruz Biotechnology	sc-200971A	DMSO
80	**N-Acetyl-L-Cysteine**	Santa Cruz Biotechnology	sc-202232	DMSO
81	**Nateglinide**	Santa Cruz Biotechnology	sc-394067A	DMSO
82	**Nirmatrelvir**	TargetMol	T9351, CAS: 2628280-40-8	DMSO
83	**Octenidine dihydrochloride**	Santa Cruz Biotechnology	sc-478739	DMSO
84	**Omeprazole**	Sigma	19329-50MG	DMSO
85	**Oseltamivir phosphate**	Sigma	SML1606	DMSO
86	**Oxymetazoline hydrochloride**	Merck	O2378	DMSO
87	**Paclitaxel**	Biomol	AG-CN2-0045-M025	DMSO
88	**Pantoprazole sodium**	Sigma	PHR1604	DMSO
89	**Penicillin sodium salt**	Santa Cruz Biotechnology	sc-257971B	DMSO
90	**Piperacillin sodium**	Santa Cruz Biotechnology	sc-205808	DMSO
91	**Prednisolone**	Santa Cruz Biotechnology	sc-205815	DMSO
92	**Propofol**	Sigma	Y0000016	DMSO
93	**Propranolol**	Santa Cruz Biotechnology	sc-3580	DMSO
94	**Pyrazinamide**	Santa Cruz Biotechnology	sc-205824A	DMSO
95	**Ramiprilat**	Santa Cruz Biotechnology	sc-212767C	DMSO
96	**Sirolimus**	Biomol	LC-R-5000_50mg	DMSO
97	**Remdesivir**	TargetMol	T7222	DMSO
99	**Rifampicin**	Merck	R3501-250MG	DMSO
100	**Ritonavir**	TargetMol	TGM-T1525-50MG	DMSO
101	**Rivaroxaban**	TargetMol	T1184-25mg-TM	DMSO
102	**Rocuronium bromide**	Sigma	Y0001766	DMSO
103	**Salmeterol xinafoate**	Santa Cruz Biotechnology	sc-202231A	DMSO

104	**Sevoflurane**	Biomol (Cayman Chemical)	Cay23996-5	DMSO
105	**SN38**	Santa Cruz Biotechnology	sc-203697	DMSO
106	**Suxamethonium chloride**	Sigma	S2200000	DMSO
107	**Tacrolimus**	Biozol (SellekChem)	SEL-S5003-50MG	DMSO
108	**Tamoxifen**	Sigma	85256	DMSO
109	**TAS102 (Trifluridine/ Tipiracil Hydrochloride)**	TargetMol	CAS 733030-01-8	DMSO
110	**Tazobactam sodium**	Santa Cruz Biotechnology	sc-205853	DMSO
111	**Tenofovir**	TargetMol	T1649	DMSO
112	**Vancomycin hydrochloride**	Santa Cruz Biotechnology	sc-204938	DMSO
113	**Warfarin**	Sigma	45706	DMSO
114	**Cephalexin monohydrate**	Santa Cruz Biotechnology	sc-487556	DMSO
115	**Norepinephrine**	Merck	A7257	DMSO
116	**Dabigatran**	TargetMol	TGM-T6295-25MG	DMSO
117	**Clavulanic acid potassium salt**	Santa Cruz Biotechnology	sc-207446	DMSO
W1	**Carboplatin**	Santa Cruz Biotechnology	sc-202093	Water
W2	**Cisplatin**	Santa Cruz Biotechnology	sc-200896	Water
W3	**Colistin sulphate salt**	Merck	C4461-100MG	Water
W4	**Enoxaparin sodium**	Sigma	E0180000	Water
W5	**Fosfomycin disodium**	Santa Cruz Biotechnology	sc-211542	Water
W6	**Gabapentin**	Sigma	PHR1049	Water
W7	**Heparin sodium**	Biozol (SellekChem)	SEL-S1346-200MG	Water
W8	**Oxaliplatin**	Biomol	AG-CR1-3592-M025	Water
W9	**Salbutamol hemisulfate**	Santa Cruz Biotechnology	sc-203373	Water
W10	**Tobramycin**	Santa Cruz Biotechnology	sc-204917A	Water
W11	**Tranexamic acid**	Santa Cruz Biotechnology	sc-204921	Water
W12	**Tramadol hydrochloride**	Sigma	42965	Water

Supplementary Table 3:

Statistical evaluation of *in vivo* experiments in *C. albicans* infected *G. mellonella.* ns = not significant, *p≤0.05, **p≤0.01, ***p≤0.001.

Compound		Calb + Drug 2	Calb + Buffer	Calb + FLC + Drug 2	Calb + FLC	Control Drug 2	Buffer control
Ethinyl Estradiol	Ca + Drug 2	-					
	Ca + Buffer	ns	-				
	Ca + FLC + Drug 2	*	ns	-			
	Ca + FLC	***	***	***	-		
	Control Drug 2	***	***	***	ns	-	
	Buffer control	***	***	***	***	ns	-
Loperamide hydrochloride	Ca + Drug 2	-					
	Ca + Buffer	ns	-				
	Ca + FLC + Drug 2	**	*	-			
	Ca + FLC	***	***	**	-		
	Control Drug 2	***	***	***	*	-	
	Buffer control	***	***	***	*	ns	-
Levothyroxine sodium	Ca + Drug 2	-					
	Ca + Buffer	ns	-				
	Ca + FLC + Drug 2	ns	ns	-			
	Ca + FLC	***	**	**	-		
	Control Drug 2	***	**	**	***	-	
	Buffer control	***	***	***	***	ns	-
Tacrolimus	Ca + Drug 2	-					

	Ca + Buffer	ns	-				
	Ca + FLC + Drug 2	***	***	-			
	Ca + FLC	***	***	ns	-		
	Control Drug 2	***	***	*	ns	-	
	Buffer control	***	***	**	*	ns	-
Carvedilol	Ca + Drug 2	-					
	Ca + Buffer	ns	-				
	Ca + FLC + Drug 2	***	**	-			
	Ca + FLC	***	***	ns	-		
	Control Drug 2	***	***	*	ns	-	
	Buffer control	***	***	***	**	ns	-
Mycophenolate	Ca + Drug 2	-					
	Ca + Buffer	ns	-				
	Ca + FLC + Drug 2	***	ns	-			
	Ca + FLC	***	ns	ns	-		
	Control Drug 2	***	**	ns	ns	-	
	Buffer control	***	**	ns	ns	ns	-
Desogestrel	Ca + Drug 2	-					
	Ca + Buffer	ns	-				
	Ca + FLC + Drug 2	***	***	-			
	Ca + FLC	***	***	ns	-	-	

	Buffer control	***	***	ns	*	-	-
Salmeterol	Ca + Drug 2	-					
	Ca + Buffer	ns	-				
	Ca + FLC + Drug 2	***	**	-			
	Ca + FLC	***	***	ns	-		
	Control Drug 2	***	***	*	ns	-	
	Buffer control	***	***	**	**	ns	-
Doxorubicin	Ca + Drug 2	-					
	Ca + Buffer	ns	-				
	Ca + FLC + Drug 2	***	ns	-			
	Ca + FLC	***	**	ns	-		
	Control Drug 2	**	*	ns	ns	-	
	Buffer control	***	***	**	**	ns	-
Dienogest	Ca + Drug 2	-					
	Ca + Buffer	ns	-				
	Ca + FLC + Drug 2	***	***	-			
	Ca + FLC	***	***	ns	-		
	Control Drug 2	***	***	*	ns	-	
	Buffer control	***	***	***	***	ns	-

2. Supplementary Figures

Supplementary Figure 1:

Growth rates of drugs that showed an effect on fungal growth in the presence of FLC or ANI, here exposed to *C. albicans* without any added antifungal (compound control). Data show a strong reduction in growth rate with both compounds that had intrinsic antifungal properties, including octenidine and sirolimus, but no impact on other hits. Growth rates were calculated based on the R package “growthcurver” and normalized to the average of the DMSO control of each plate (*C. albicans* growth only). N=3 technical replicates. _w = drug was solved in water (instead of DMSO).

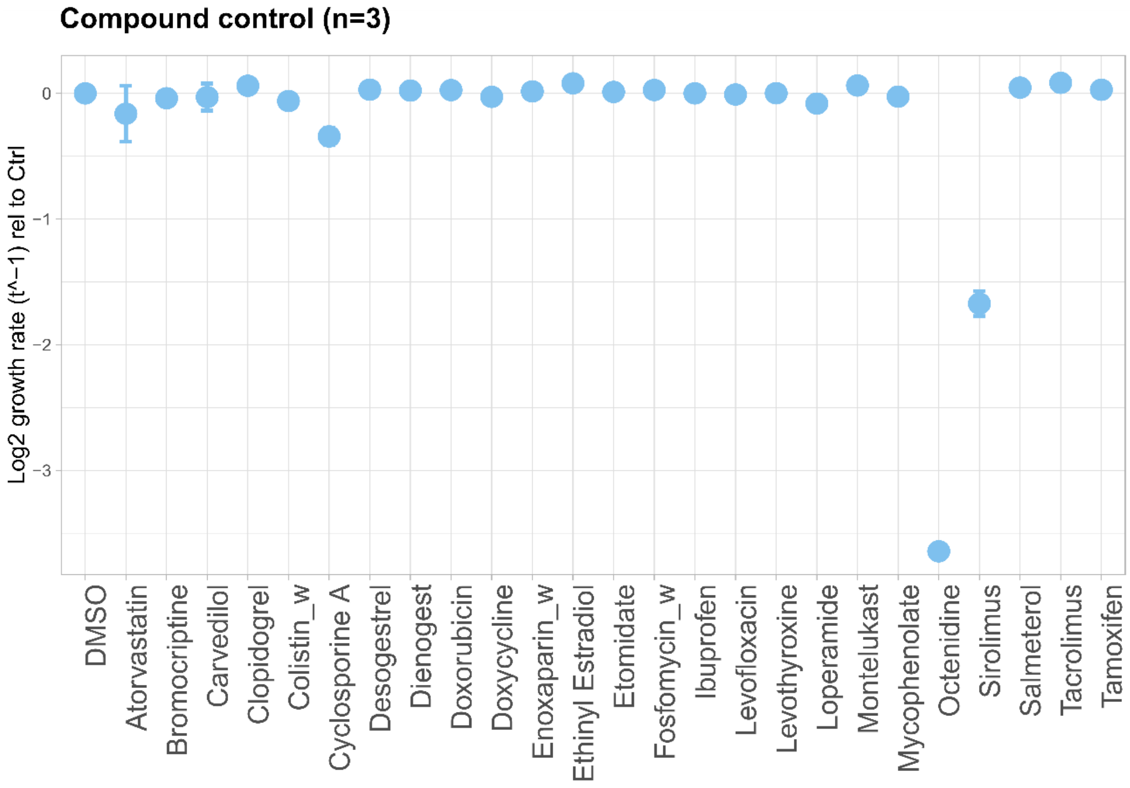

Supplementary Figure 2

Targeted drug testing with FLC **(A)** and ANI **(B)**. To provide a dynamic range of drug concentrations that would detect both improvement and attenuation of the FLC and ANI activities, we tested various concentrations of antifungals at i) below MIC50 (0.375 µG/ mL FLC and 0.008 µG/ mL ANI), ii) beyond MIC50 (0.5 µG/ mL and 1.0 µG/ mL FLC as well as 0.02 µG/ mL and 0.04 µG/ mL ANI) concentrations and iii) without an antifungal as a control for compounds potentially acting alone on *C. albicans.* The grey dashed line shows the threshold for a concerned "hit", having a mean of the fungal biomass > 1.5x or <0.5x OD600 of the mean of the REF control (antifungal + DMSO of the matched condition. Controls are shown in grey.

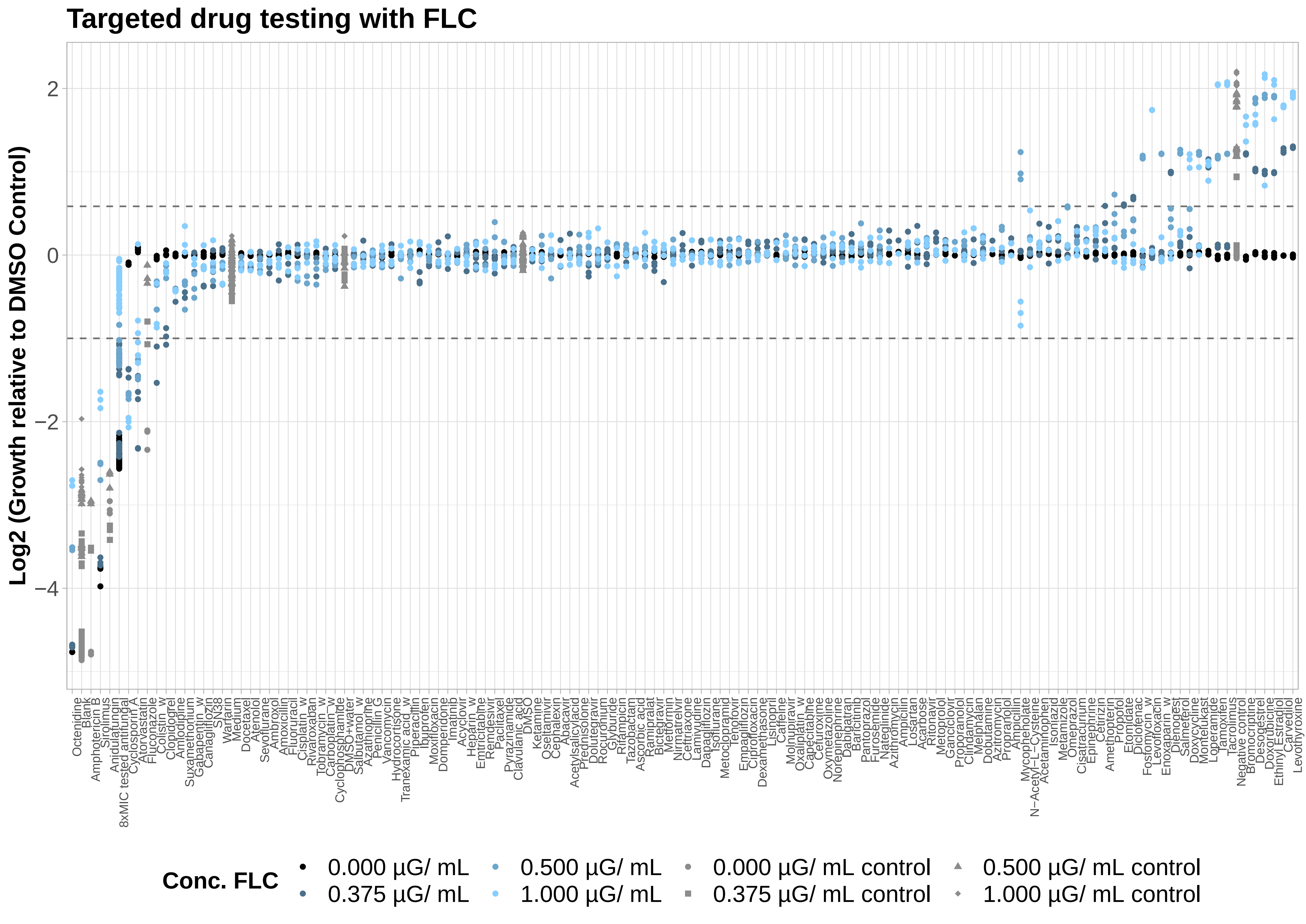

Targeted drug testing with FLC
Log2 (Growth relative to DMSO Control)
2
0
−2
−4
Octenidine
Blank
Amphotericin B
Sirolimus
8xMIC tested antifungal
Anidulafungin
Cyclosporin A
Atorvastatin
Fluconazole
Colistin_w
Clopidogrel
Amlodipine
Suxamethonium
Gabapentin_w
Canagliflozin
SN38
Warfarin
Medium
Docetaxel
Atenolol
Sevoflurane
Ambroxol
Amoxicillin
Fluoruracil
Cisplatin_w
Rivaroxaban
Tobramycin_w
Carboplatin_w
Cyclophosphamide
DMSO+water
Salbutamol_w
Azathioprine
Penicillin G
Vancomycin
Hydrocortisone
Tranexamic acid_w
Piperacillin
Ibuprofen
Moxifloxacin
Domperidone
Imatinib
Acyclovir
Heparin_w
Emtricitabine
Remdesivir
Paclitaxel
Pyrazinamide
Clavulanic acid
DMSO
Ketamine
Oseltamivir
Cephalexin
Abacavir
Acetylsalicylacid
Prednisolone
Dolutegravir
Rocuronium
Glyburide
Rifampicin
Tazobactam
Ascorbic acid
Ramipralat
Bictegravir
Metformin
Nirmatrelvir
Ceftriaxone
Lamivudine
Dapagliflozin
Isoflurane
Metoclopramid
Tenofovir
Empagliflozin
Ciprofloxacin
Dexamethasone
Lisinopril
Caffeine
Molnupiravir
Oxaliplatin_w
Capecitabine
Cefuroxime
Oxymetazoline
Norepinephrine
Dabigatran
Baricitinib
Pantoprazol
Furosemide
Nateglinide
Azithromycin
Ampicilin
Losartan
Acarbose
Ritonavir
Metoprolol
Ganciclovir
Proporanolol
Clindamycin
Melphalan
Dobutamine
Azitromycin
Propranolol
Ampicillin
Mycophenolate
N−Acetyl−L−Cysteine
Acetaminophen
Isoniazid
Metamizole
Omeprazol
Cisatracurium
Epinephrine
Cetirizin
Amethopterin
Propofol
Etomidate
Diclofenac
Fosfomycin_w
Levofloxacin
Enoxaparin_w
Dienogest
Salmeterol
Doxycycline
Montelukast
Loperamide
Tamoxifen
Tacrolimus
Negative control
Bromocriptine
Desogestrel
Doxorubicine
Ethinyl Estradiol
Carvedilol
Levothyroxine
Conc. FLC
0.000 µG/ mL
0.375 µG/ mL
0.500 µG/ mL
1.000 µG/ mL
0.000 µG/ mL control
0.375 µG/ mL control
0.500 µG/ mL control
1.000 µG/ mL control

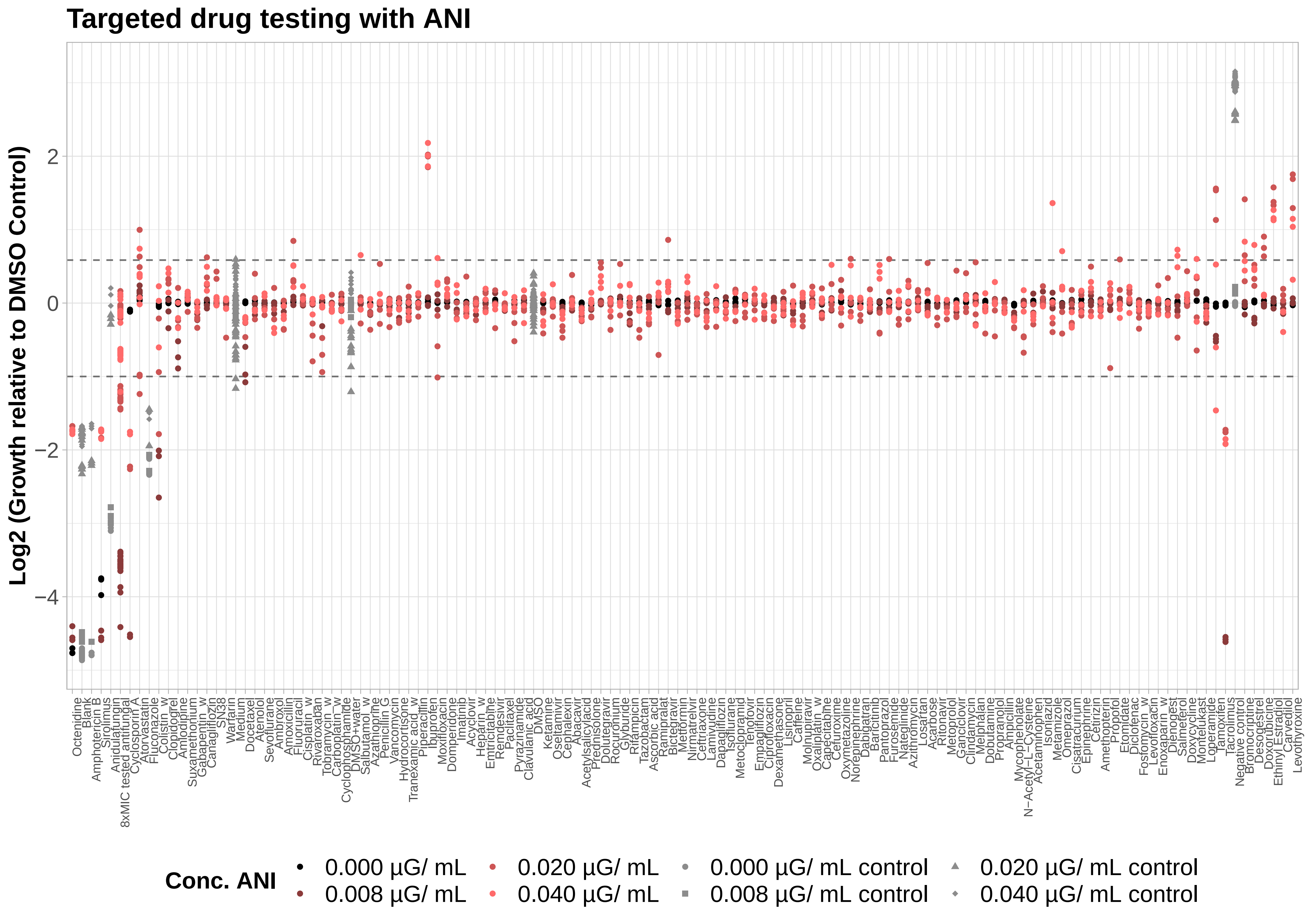

Targeted drug testing with ANI
Log2 (Growth relative to DMSO Control)
2
0
−2
−4
Octenidine
Blank
Amphotericin B
Sirolimus
Anidulafungin
8xMIC tested antifungal
Cyclosporin A
Atorvastatin
Fluconazole
Colistin_w
Clopidogrel
Amlodipine
Suxamethonium
Gabapentin_w
Canagliflozin
SN38
Warfarin
Medium
Docetaxel
Atenolol
Sevoflurane
Ambroxol
Amoxicillin
Fluoruracil
Cisplatin_w
Rivaroxaban
Tobramycin_w
Carboplatin_w
Cyclophosphamide
DMSO+water
Salbutamol_w
Azathioprine
Penicillin G
Vancomycin
Hydrocortisone
Tranexamic acid_w
Piperacillin
Ibuprofen
Moxifloxacin
Domperidone
Imatinib
Acyclovir
Heparin_w
Emtricitabine
Remdesivir
Paclitaxel
Pyrazinamide
Clavulanic acid
DMSO
Ketamine
Oseltamivir
Cephalexin
Abacavir
Acetylsalicylacid
Prednisolone
Dolutegravir
Rocuronium
Glyburide
Rifampicin
Tazobactam
Ascorbic acid
Ramipralat
Bictegravir
Metformin
Nirmatrelvir
Ceftriaxone
Lamivudine
Dapagliflozin
Isoflurane
Metoclopramid
Tenofovir
Empagliflozin
Ciprofloxacin
Dexamethasone
Lisinopril
Caffeine
Molnupiravir
Oxaliplatin_w
Capecitabine
Cefuroxime
Oxymetazoline
Norepinephrine
Dabigatran
Baricitinib
Pantoprazol
Furosemide
Nateglinide
Azithromycin
Losartan
Acarbose
Ritonavir
Metoprolol
Ganciclovir
Clindamycin
Melphalan
Dobutamine
Propranolol
Ampicillin
Mycophenolate
N−Acetyl−L−Cysteine
Acetaminophen
Isoniazid
Metamizole
Omeprazol
Cisatracurium
Epinephrine
Cetirizin
Amethopterin
Propofol
Etomidate
Diclofenac
Fosfomycin_w
Levofloxacin
Enoxaparin_w
Dienogest
Salmeterol
Doxycycline
Montelukast
Loperamide
Tamoxifen
Tacrolimus
Negative control
Bromocriptine
Desogestrel
Doxorubicine
Ethinyl Estradiol
Carvedilol
Levothyroxine
Conc. ANI
0.000 µG/ mL
0.008 µG/ mL
0.020 µG/ mL
0.040 µG/ mL
0.000 µG/ mL control
0.008 µG/ mL control
0.020 µG/ mL control
0.040 µG/ mL control

Supplementary Figure 3

Checkerboard assays with FLC (blue) and ANI (red).

a) Drugs that suppressed the fungal biomass in tageted drug tests with FLC (blue) or ANI (red).

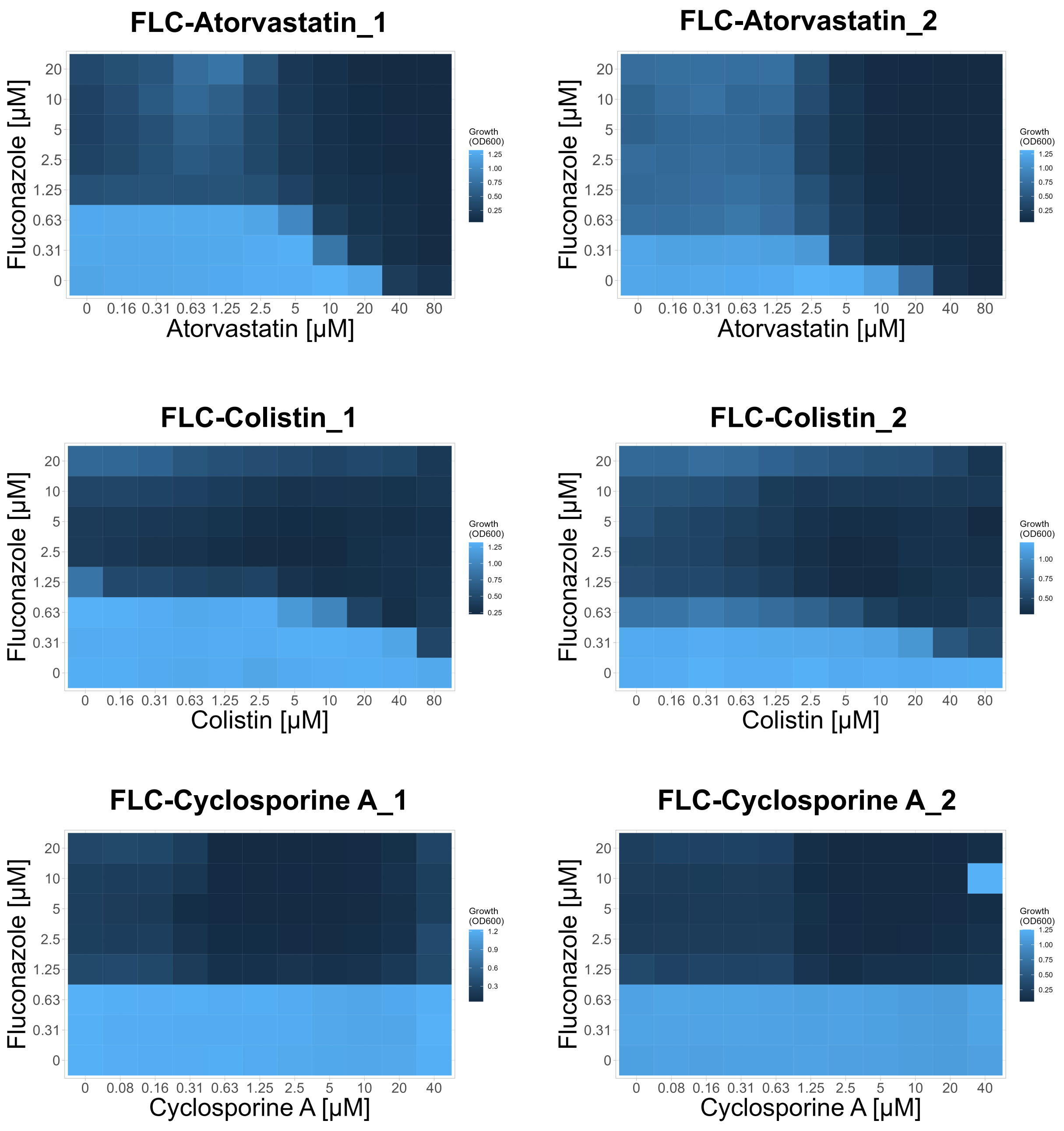

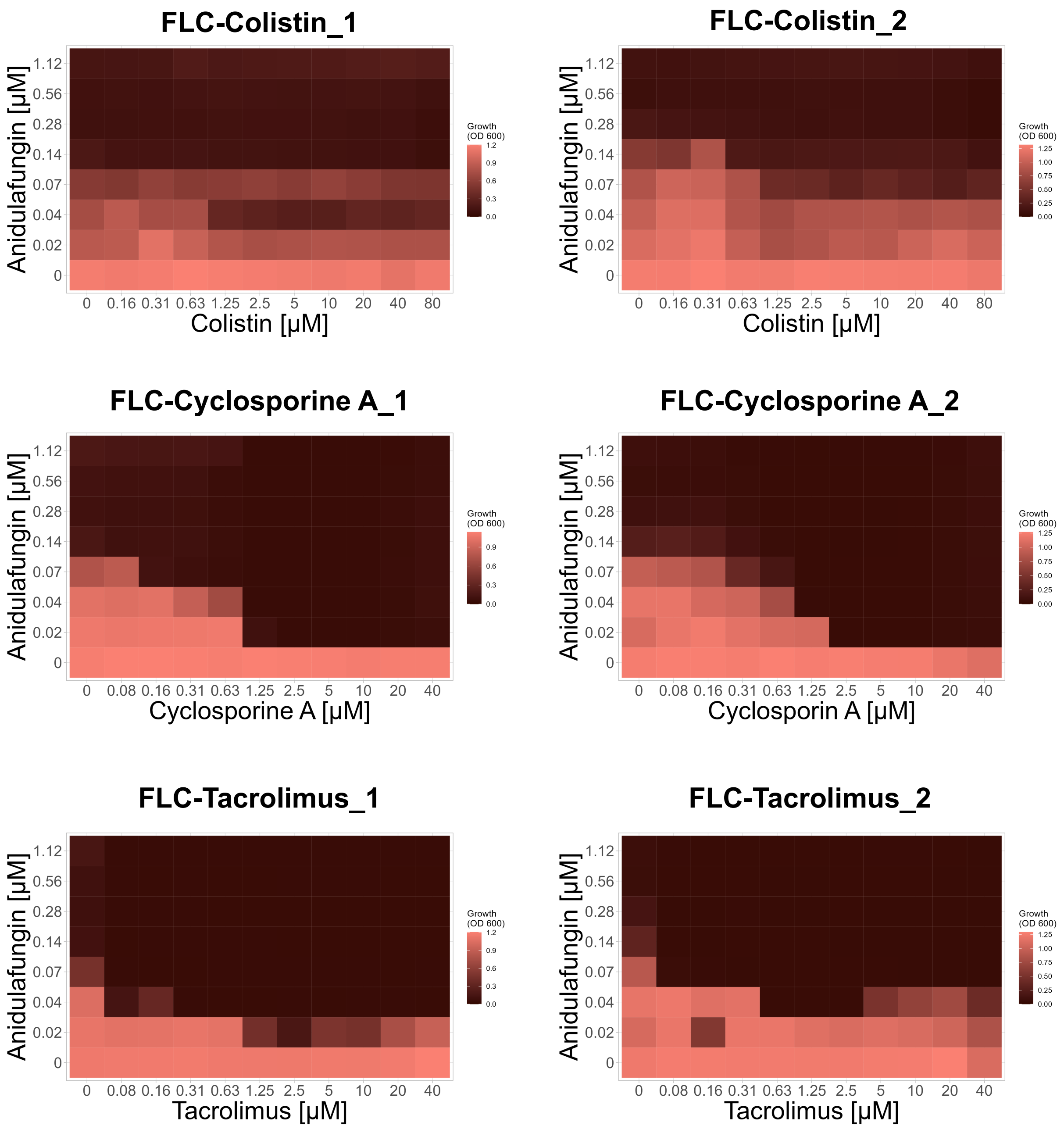
FLC-Colistin_1
FLC-Colistin_2
FLC-Cyclosporine A_1
FLC-Cyclosporine A_2
FLC-Tacrolimus_1
FLC-Tacrolimus_2
Anidulafungin [µM]
1.12
0.56
0.28
0.14
0.07
0.04
0.02
0
Colistin [µM]
0 0.16 0.31 0.63 1.25 2.5 5 10 20 40 80
Cyclosporine A [µM]
Cyclosporin A [µM]
Tacrolimus [µM]
0 0.08 0.16 0.31 0.63 1.25 2.5 5 10 20 40
Growth (OD 600)
1.2 0.9 0.6 0.3 0.0
1.25 1.00 0.75 0.50 0.25 0.00

b) Drugs that increased the fungal biomass in tageted drug tests with FLC (blue) or ANI (red).

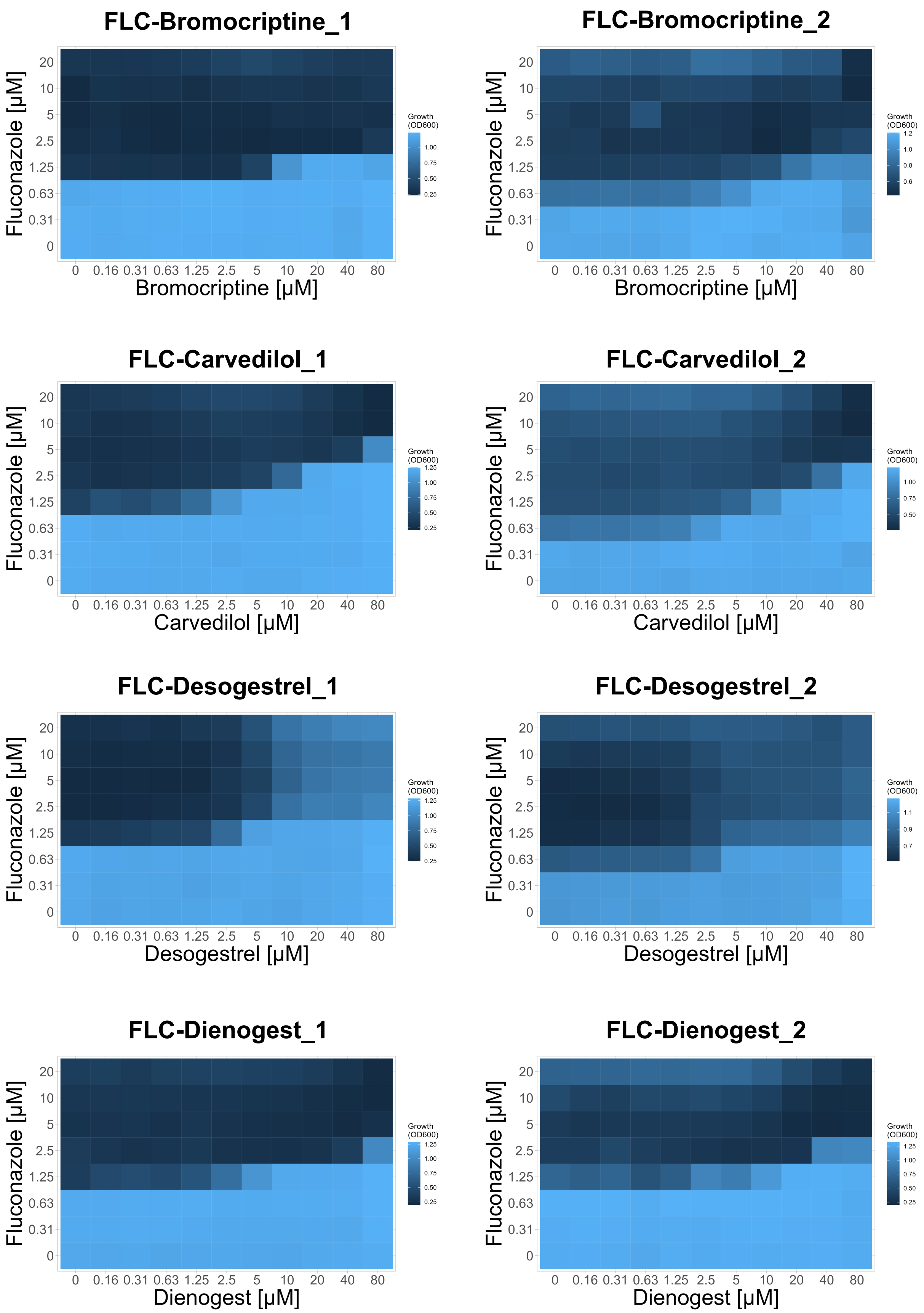

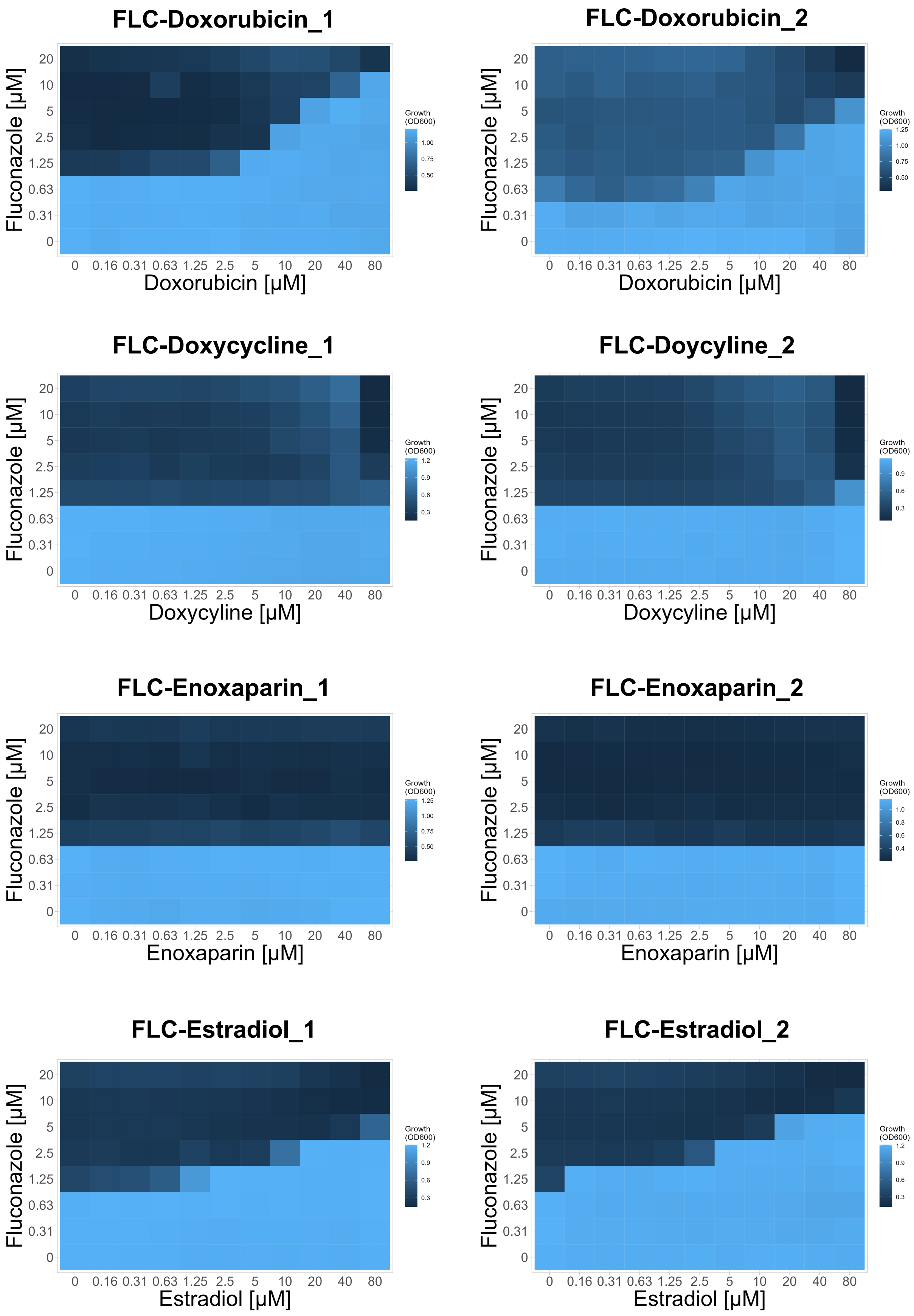
FLC-Doxorubicin_1
FLC-Doxorubicin_2
Fluconazole [μM]
20
10
5
2.5
1.25
0.63
0.31
0
0 0.16 0.31 0.63 1.25 2.5 5 10 20 40 80
Doxorubicin [μM]
Growth (OD600)
FLC-Doxycycline_1
FLC-Doycyline_2
Doxycyline [μM]
FLC-Enoxaparin_1
FLC-Enoxaparin_2
Enoxaparin [μM]
FLC-Estradiol_1
FLC-Estradiol_2
Estradiol [μM]

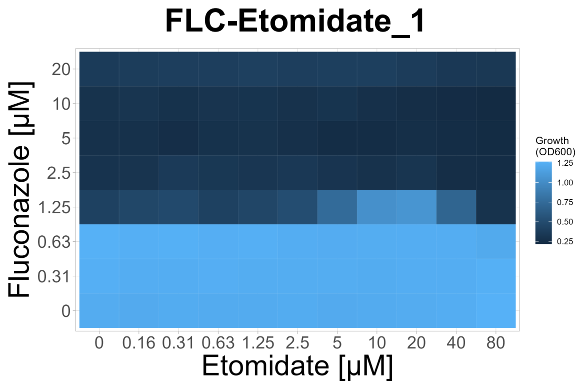
FLC-Etomidate_1
Fluconazole [μM]
20 10 5 2.5 1.25 0.63 0.31 0
0 0.16 0.31 0.63 1.25 2.5 5 10 20 40 80
Etomidate [μM]
Growth (OD600)
1.25 1.00 0.75 0.50 0.25

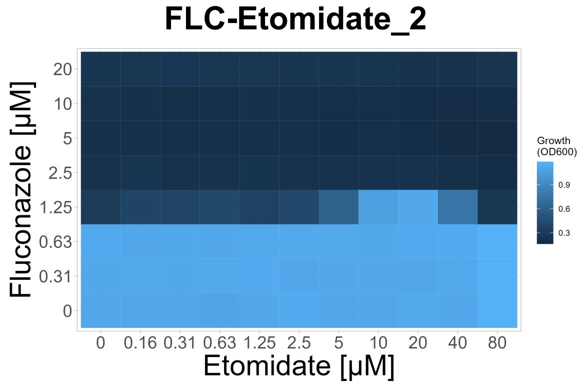
FLC-Etomidate_2
Fluconazole [μM]
20 10 5 2.5 1.25 0.63 0.31 0
0 0.16 0.31 0.63 1.25 2.5 5 10 20 40 80
Etomidate [μM]
Growth (OD600)
0.9 0.6 0.3

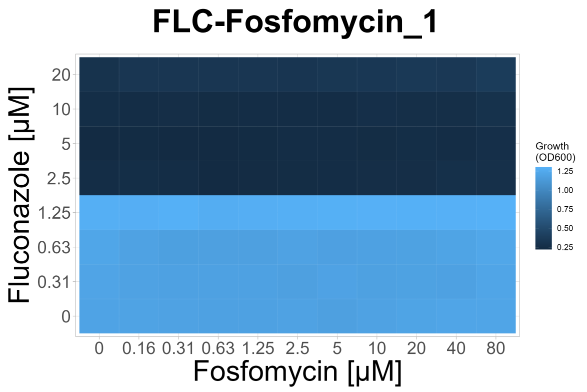
FLC-Fosfomycin_1
Fluconazole [μM]
20 10 5 2.5 1.25 0.63 0.31 0
0 0.16 0.31 0.63 1.25 2.5 5 10 20 40 80
Fosfomycin [μM]
Growth (OD600)
1.25 1.00 0.75 0.50 0.25

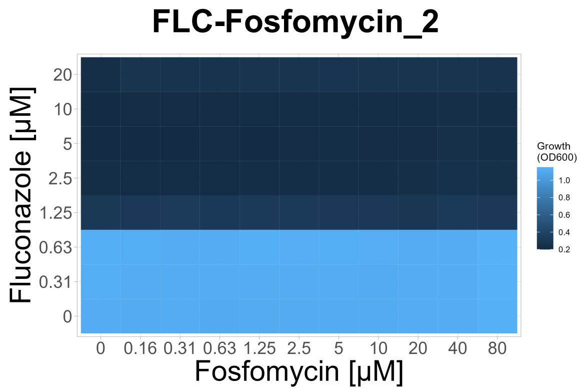
FLC-Fosfomycin_2
Fluconazole [μM]
20 10 5 2.5 1.25 0.63 0.31 0
0 0.16 0.31 0.63 1.25 2.5 5 10 20 40 80
Fosfomycin [μM]
Growth (OD600)
1.0 0.8 0.6 0.4 0.2

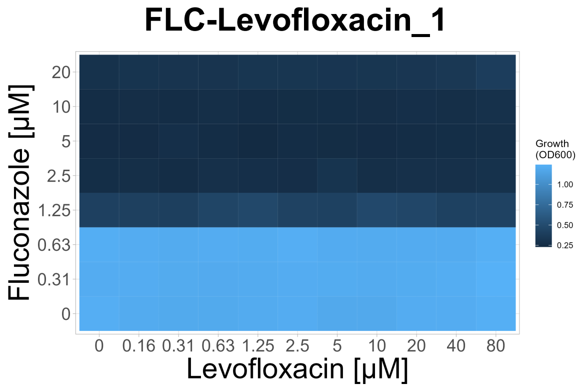
FLC-Levofloxacin_1
Fluconazole [μM]
20 10 5 2.5 1.25 0.63 0.31 0
0 0.16 0.31 0.63 1.25 2.5 5 10 20 40 80
Levofloxacin [μM]
Growth (OD600)
1.00 0.75 0.50 0.25

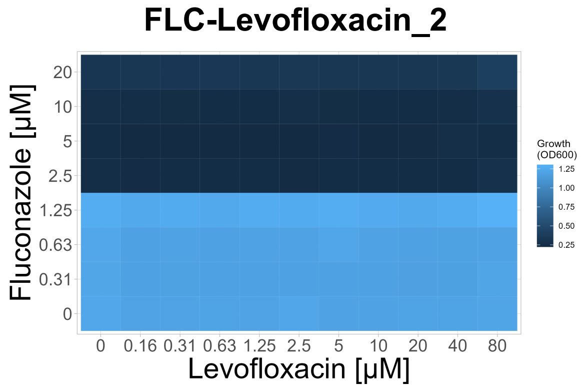
FLC-Levofloxacin_2
Fluconazole [μM]
20 10 5 2.5 1.25 0.63 0.31 0
0 0.16 0.31 0.63 1.25 2.5 5 10 20 40 80
Levofloxacin [μM]
Growth (OD600)
1.25 1.00 0.75 0.50 0.25

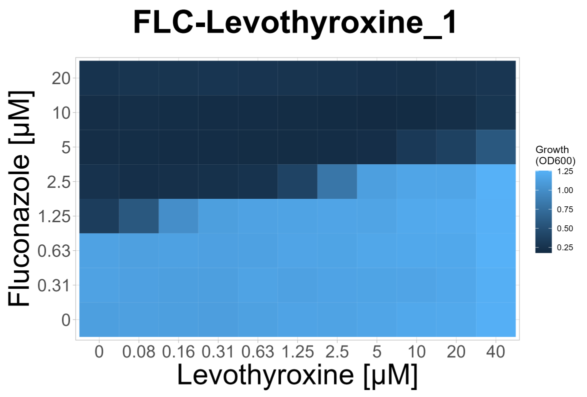
FLC-Levothyroxine_1
Fluconazole [μM]
20 10 5 2.5 1.25 0.63 0.31 0
0 0.08 0.16 0.31 0.63 1.25 2.5 5 10 20 40
Levothyroxine [μM]
Growth (OD600)
1.25 1.00 0.75 0.50 0.25

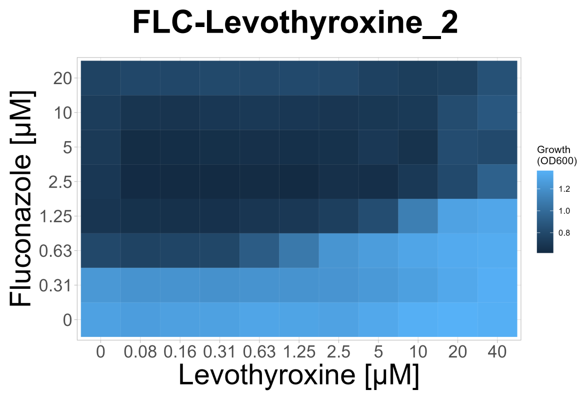
FLC-Levothyroxine_2
Fluconazole [μM]
20 10 5 2.5 1.25 0.63 0.31 0
0 0.08 0.16 0.31 0.63 1.25 2.5 5 10 20 40
Levothyroxine [μM]
Growth (OD600)
1.2 1.0 0.8

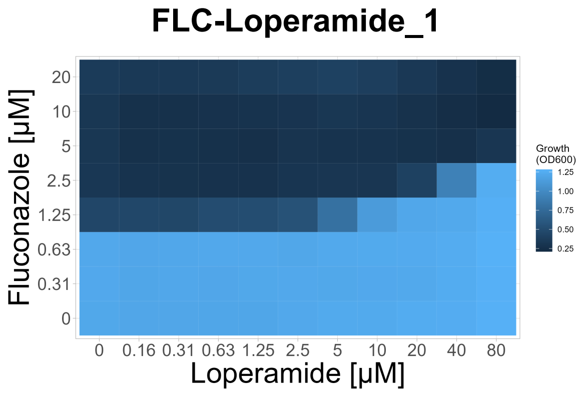
FLC-Loperamide_1
Fluconazole [µM]
20
10
5
2.5
1.25
0.63
0.31
0
0 0.16 0.31 0.63 1.25 2.5 5 10 20 40 80
Loperamide [µM]
Growth (OD600)
1.25
1.00
0.75
0.50
0.25

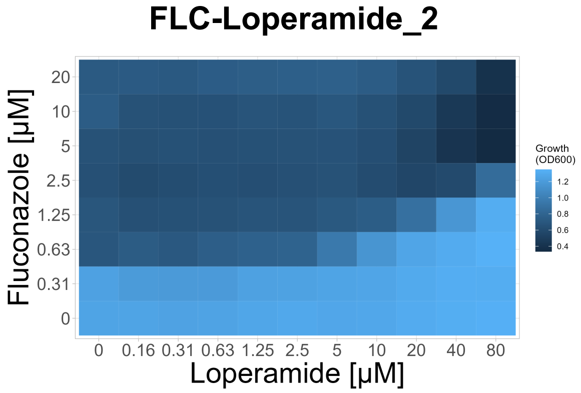
FLC-Loperamide_2
Fluconazole [µM]
20
10
5
2.5
1.25
0.63
0.31
0
0 0.16 0.31 0.63 1.25 2.5 5 10 20 40 80
Loperamide [µM]
Growth (OD600)
1.2
1.0
0.8
0.6
0.4

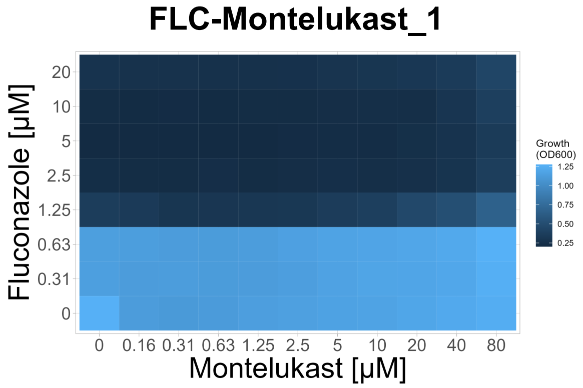
FLC-Montelukast_1
Fluconazole [µM]
20
10
5
2.5
1.25
0.63
0.31
0
0 0.16 0.31 0.63 1.25 2.5 5 10 20 40 80
Montelukast [µM]
Growth (OD600)
1.25
1.00
0.75
0.50
0.25

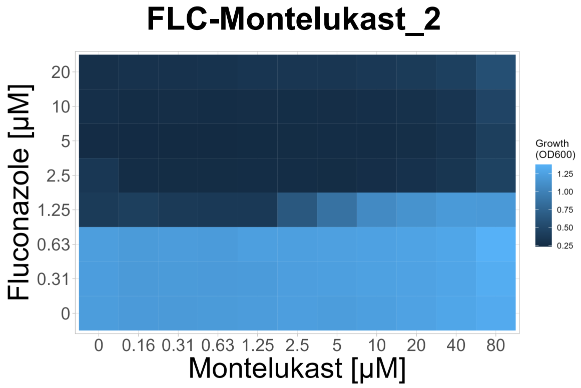
FLC-Montelukast_2
Fluconazole [µM]
20
10
5
2.5
1.25
0.63
0.31
0
0 0.16 0.31 0.63 1.25 2.5 5 10 20 40 80
Montelukast [µM]
Growth (OD600)
1.25
1.00
0.75
0.50
0.25

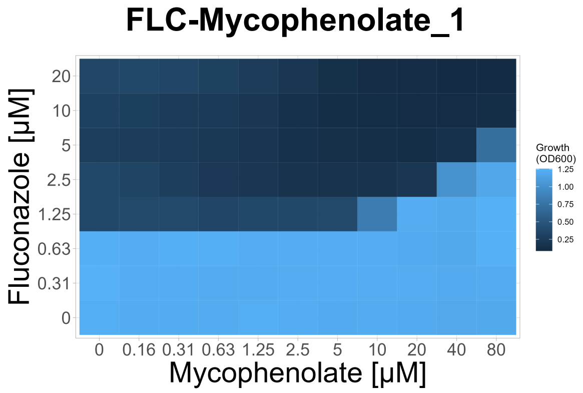
FLC-Mycophenolate_1
Fluconazole [µM]
20
10
5
2.5
1.25
0.63
0.31
0
0 0.16 0.31 0.63 1.25 2.5 5 10 20 40 80
Mycophenolate [µM]
Growth (OD600)
1.25
1.00
0.75
0.50
0.25

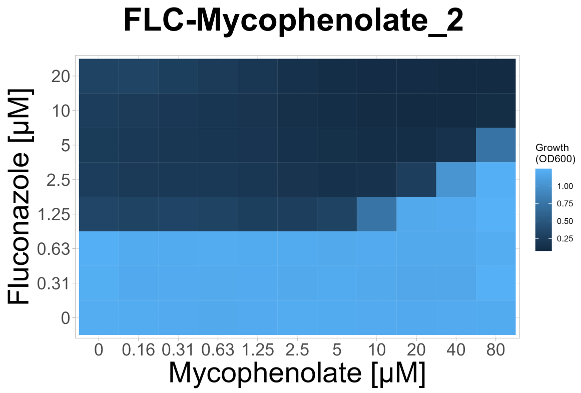
FLC-Mycophenolate_2
Fluconazole [µM]
20
10
5
2.5
1.25
0.63
0.31
0
0 0.16 0.31 0.63 1.25 2.5 5 10 20 40 80
Mycophenolate [µM]
Growth (OD600)
1.00
0.75
0.50
0.25

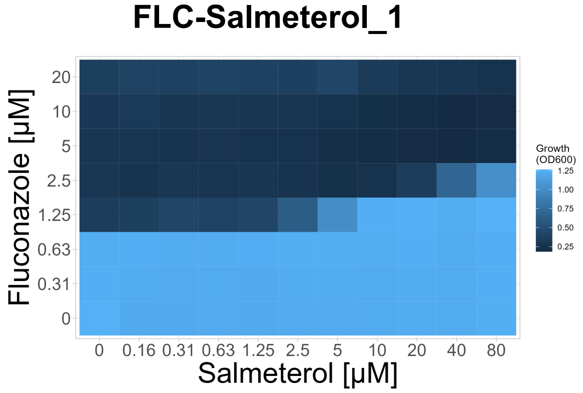
FLC-Salmeterol_1
Fluconazole [µM]
20
10
5
2.5
1.25
0.63
0.31
0
0 0.16 0.31 0.63 1.25 2.5 5 10 20 40 80
Salmeterol [µM]
Growth (OD600)
1.25
1.00
0.75
0.50
0.25

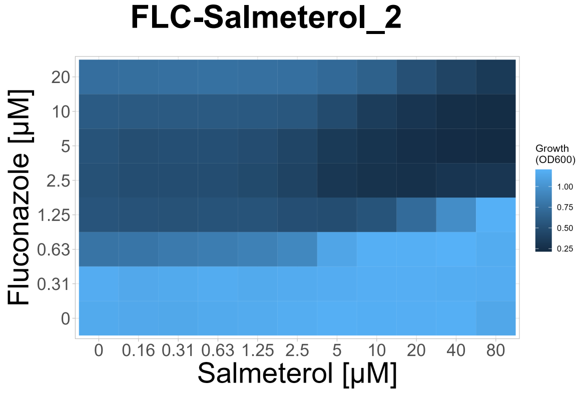
FLC-Salmeterol_2
Fluconazole [µM]
20
10
5
2.5
1.25
0.63
0.31
0
0 0.16 0.31 0.63 1.25 2.5 5 10 20 40 80
Salmeterol [µM]
Growth (OD600)
1.00
0.75
0.50
0.25

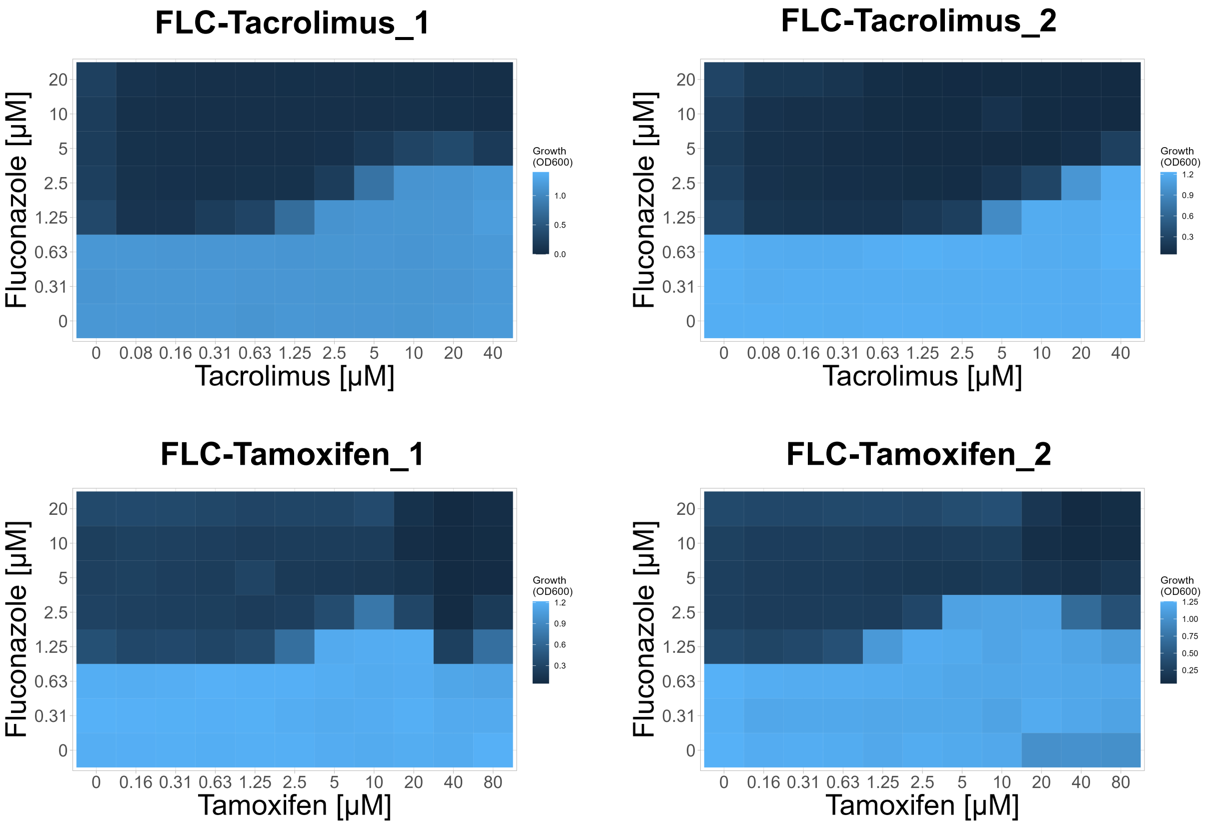
FLC-Tacrolimus_1
FLC-Tacrolimus_2
FLC-Tamoxifen_1
FLC-Tamoxifen_2
Fluconazole [μM]
Tacrolimus [μM]
Tamoxifen [μM]
Growth (OD600)
20
10
5
2.5
1.25
0.63
0.31
0
0 0.08 0.16 0.31 0.63 1.25 2.5 5 10 20 40
0 0.16 0.31 0.63 1.25 2.5 5 10 20 40 80

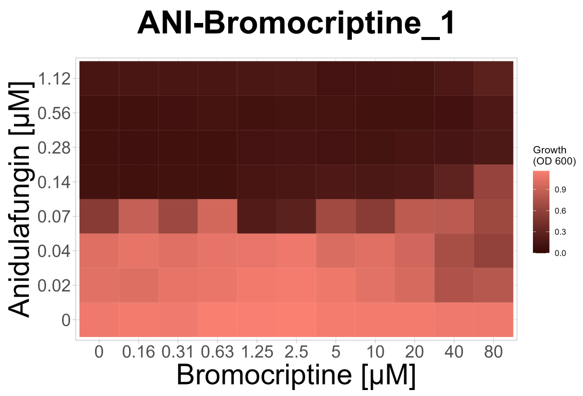
ANI-Bromocriptine_1
Anidulafungin [µM]
1.12 0.56 0.28 0.14 0.07 0.04 0.02 0
0 0.16 0.31 0.63 1.25 2.5 5 10 20 40 80
Bromocriptine [µM]
Growth (OD 600)
0.9 0.6 0.3 0.0

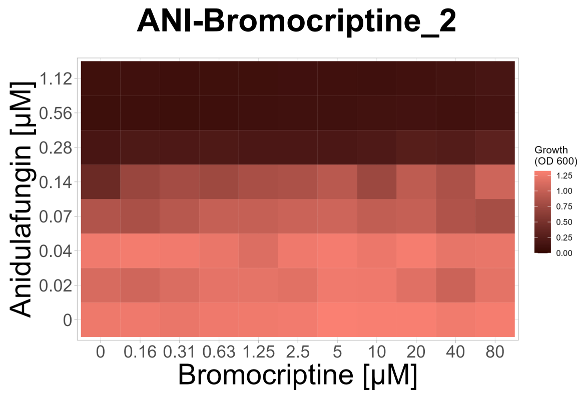
ANI-Bromocriptine_2
Anidulafungin [µM]
1.12 0.56 0.28 0.14 0.07 0.04 0.02 0
0 0.16 0.31 0.63 1.25 2.5 5 10 20 40 80
Bromocriptine [µM]
Growth (OD 600)
1.25 1.00 0.75 0.50 0.25 0.00

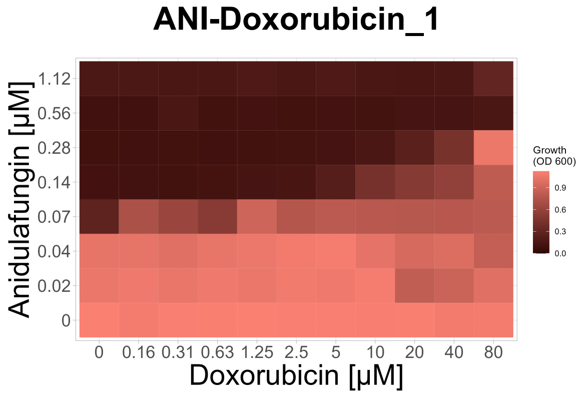
ANI-Doxorubicin_1
Anidulafungin [µM]
1.12 0.56 0.28 0.14 0.07 0.04 0.02 0
0 0.16 0.31 0.63 1.25 2.5 5 10 20 40 80
Doxorubicin [µM]
Growth (OD 600)
0.9 0.6 0.3 0.0

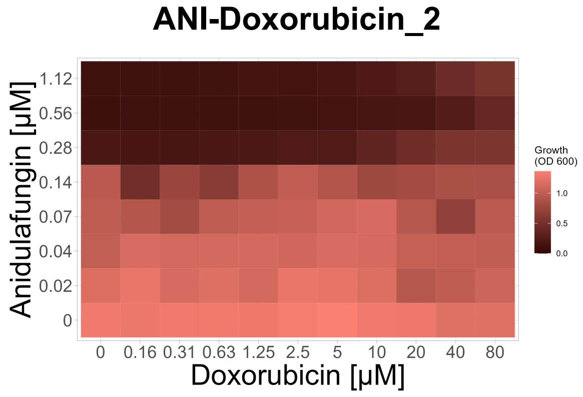
ANI-Doxorubicin_2
Anidulafungin [µM]
1.12 0.56 0.28 0.14 0.07 0.04 0.02 0
0 0.16 0.31 0.63 1.25 2.5 5 10 20 40 80
Doxorubicin [µM]
Growth (OD 600)
1.0 0.5 0.0

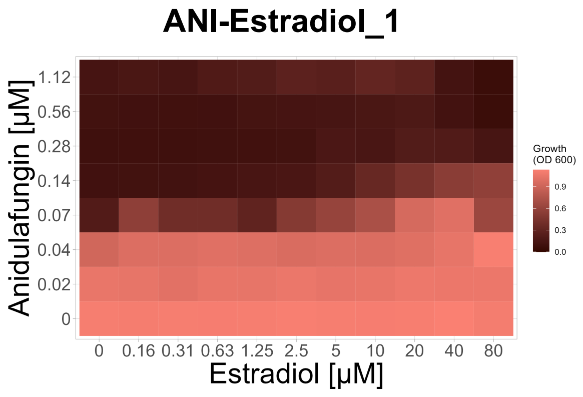
ANI-Estradiol_1
Anidulafungin [µM]
1.12 0.56 0.28 0.14 0.07 0.04 0.02 0
0 0.16 0.31 0.63 1.25 2.5 5 10 20 40 80
Estradiol [µM]
Growth (OD 600)
0.9 0.6 0.3 0.0

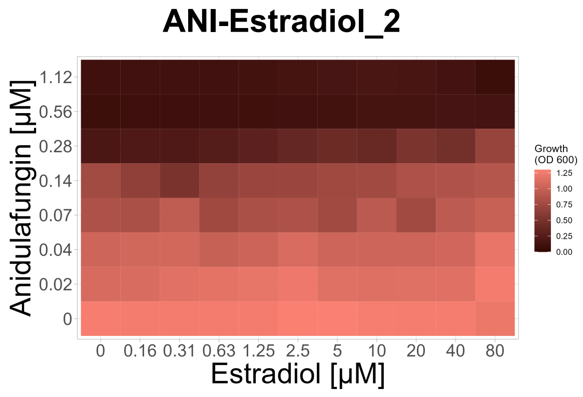
ANI-Estradiol_2
Anidulafungin [µM]
1.12 0.56 0.28 0.14 0.07 0.04 0.02 0
0 0.16 0.31 0.63 1.25 2.5 5 10 20 40 80
Estradiol [µM]
Growth (OD 600)
1.25 1.00 0.75 0.50 0.25 0.00

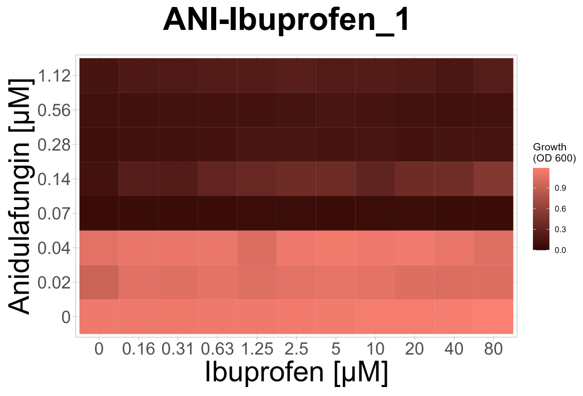
ANI-Ibuprofen_1
Anidulafungin [µM]
1.12 0.56 0.28 0.14 0.07 0.04 0.02 0
0 0.16 0.31 0.63 1.25 2.5 5 10 20 40 80
Ibuprofen [µM]
Growth (OD 600)
0.9 0.6 0.3 0.0

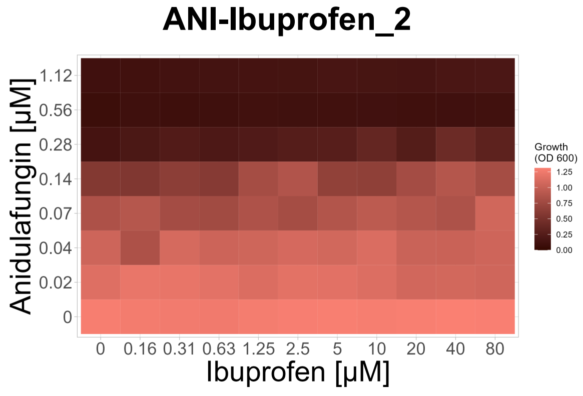
ANI-Ibuprofen_2
Anidulafungin [µM]
1.12 0.56 0.28 0.14 0.07 0.04 0.02 0
0 0.16 0.31 0.63 1.25 2.5 5 10 20 40 80
Ibuprofen [µM]
Growth (OD 600)
1.25 1.00 0.75 0.50 0.25 0.00

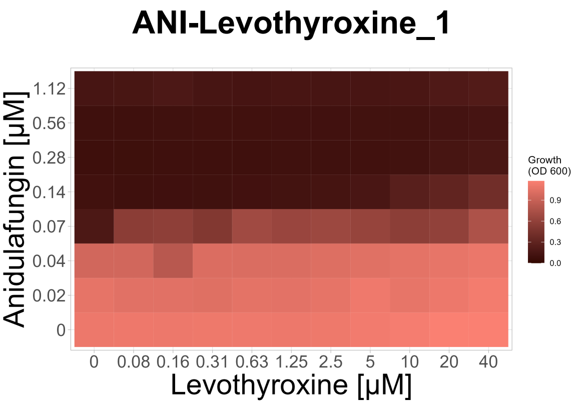
ANI-Levothyroxine_1
Anidulafungin [µM]
1.12
0.56
0.28
0.14
0.07
0.04
0.02
0
0 0.08 0.16 0.31 0.63 1.25 2.5 5 10 20 40
Levothyroxine [µM]
Growth (OD 600)
0.9
0.6
0.3
0.0

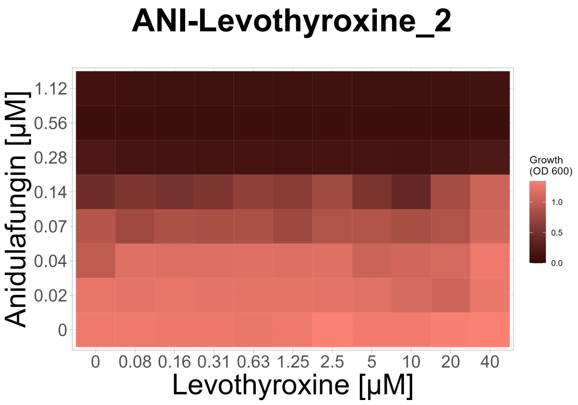
ANI-Levothyroxine_2
Anidulafungin [µM]
1.12
0.56
0.28
0.14
0.07
0.04
0.02
0
0 0.08 0.16 0.31 0.63 1.25 2.5 5 10 20 40
Levothyroxine [µM]
Growth (OD 600)
1.0
0.5
0.0

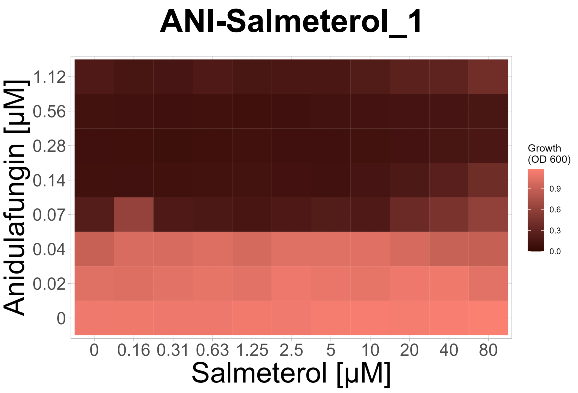
ANI-Salmeterol_1
Anidulafungin [µM]
1.12
0.56
0.28
0.14
0.07
0.04
0.02
0
0 0.16 0.31 0.63 1.25 2.5 5 10 20 40 80
Salmeterol [µM]
Growth (OD 600)
0.9
0.6
0.3
0.0

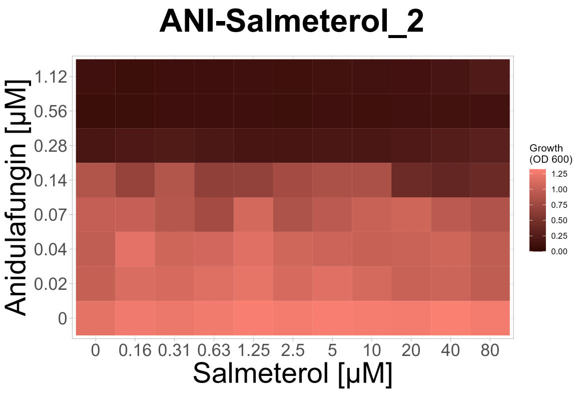
ANI-Salmeterol_2
Anidulafungin [µM]
1.12
0.56
0.28
0.14
0.07
0.04
0.02
0
0 0.16 0.31 0.63 1.25 2.5 5 10 20 40 80
Salmeterol [µM]
Growth (OD 600)
1.25
1.00
0.75
0.50
0.25
0.00

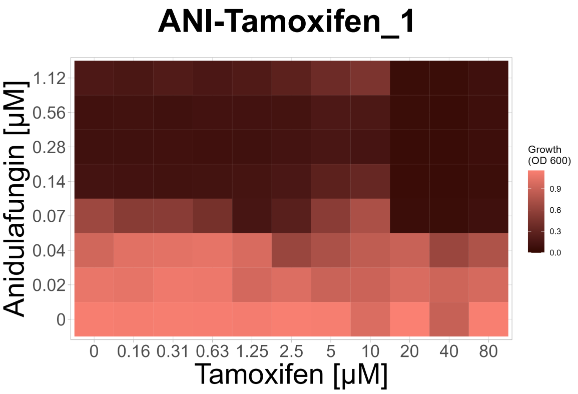
ANI-Tamoxifen_1
Anidulafungin [µM]
1.12
0.56
0.28
0.14
0.07
0.04
0.02
0
0 0.16 0.31 0.63 1.25 2.5 5 10 20 40 80
Tamoxifen [µM]
Growth (OD 600)
0.9
0.6
0.3
0.0

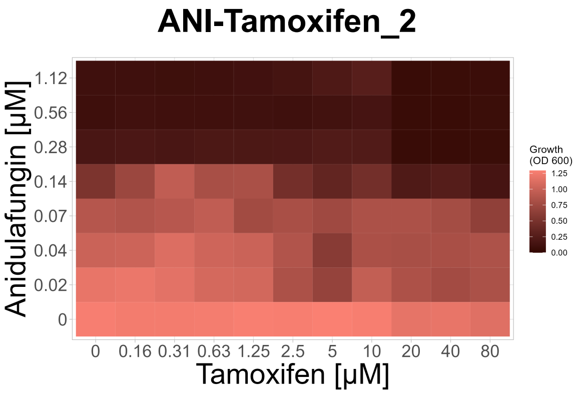
ANI-Tamoxifen_2
Anidulafungin [µM]
1.12
0.56
0.28
0.14
0.07
0.04
0.02
0
0 0.16 0.31 0.63 1.25 2.5 5 10 20 40 80
Tamoxifen [µM]
Growth (OD 600)
1.25
1.00
0.75
0.50
0.25
0.00

Supplementary Figure 4:
Growth restoration of negative interactions with FLC (blue) and ANI (red) compared to the untreated control.

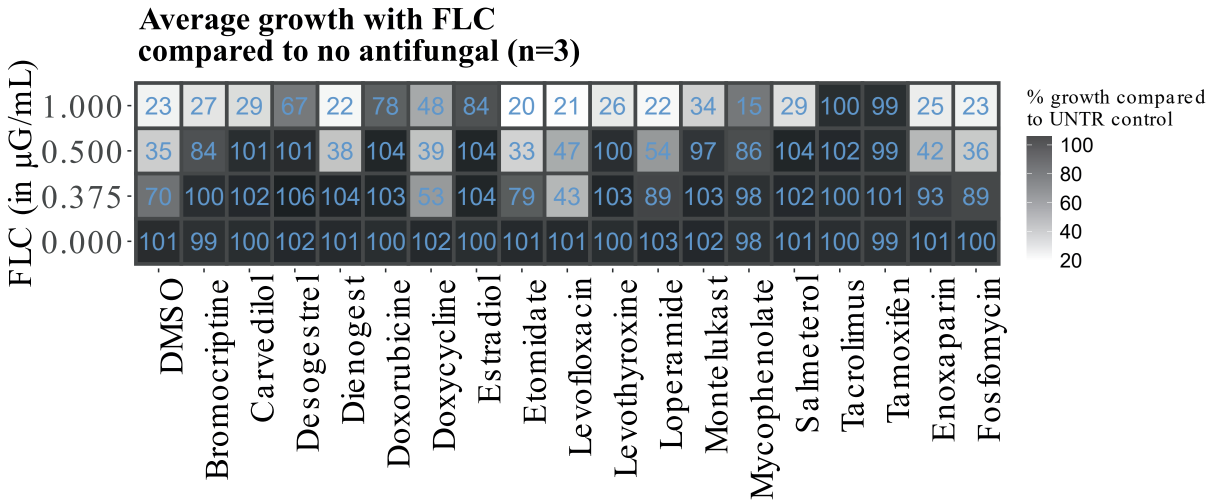

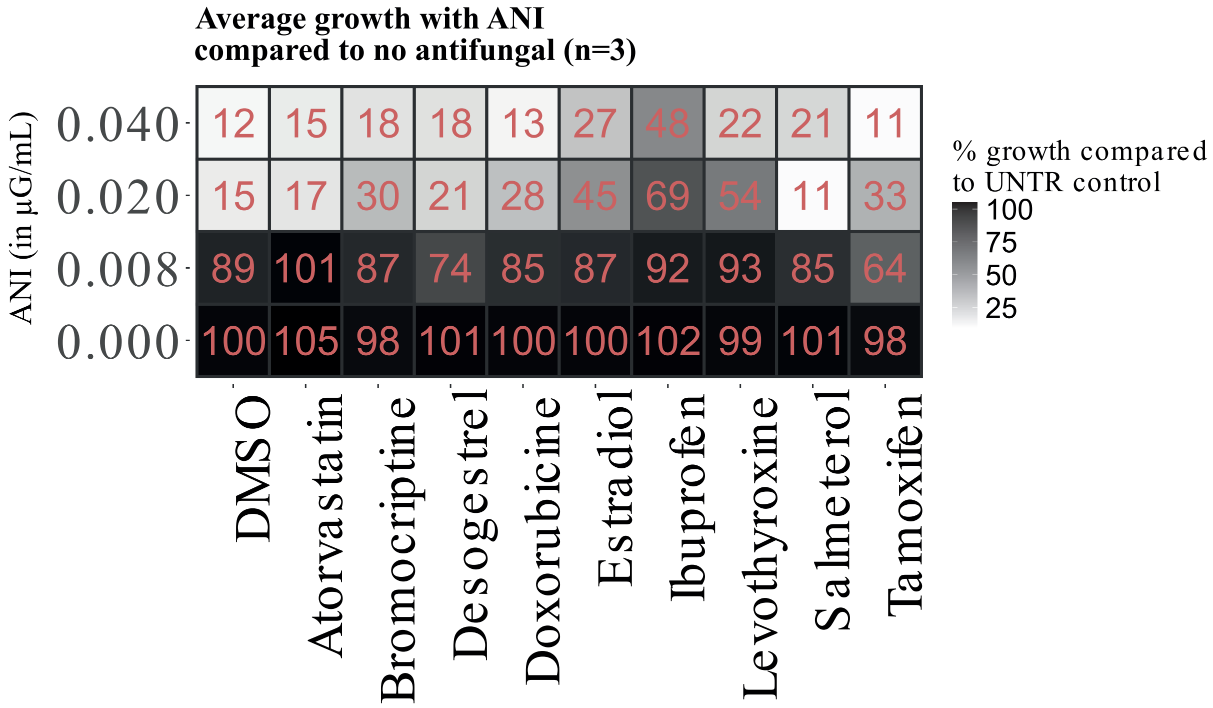

Supplementary Figure 5:

Disk diffusion assays with FLC and ANI.

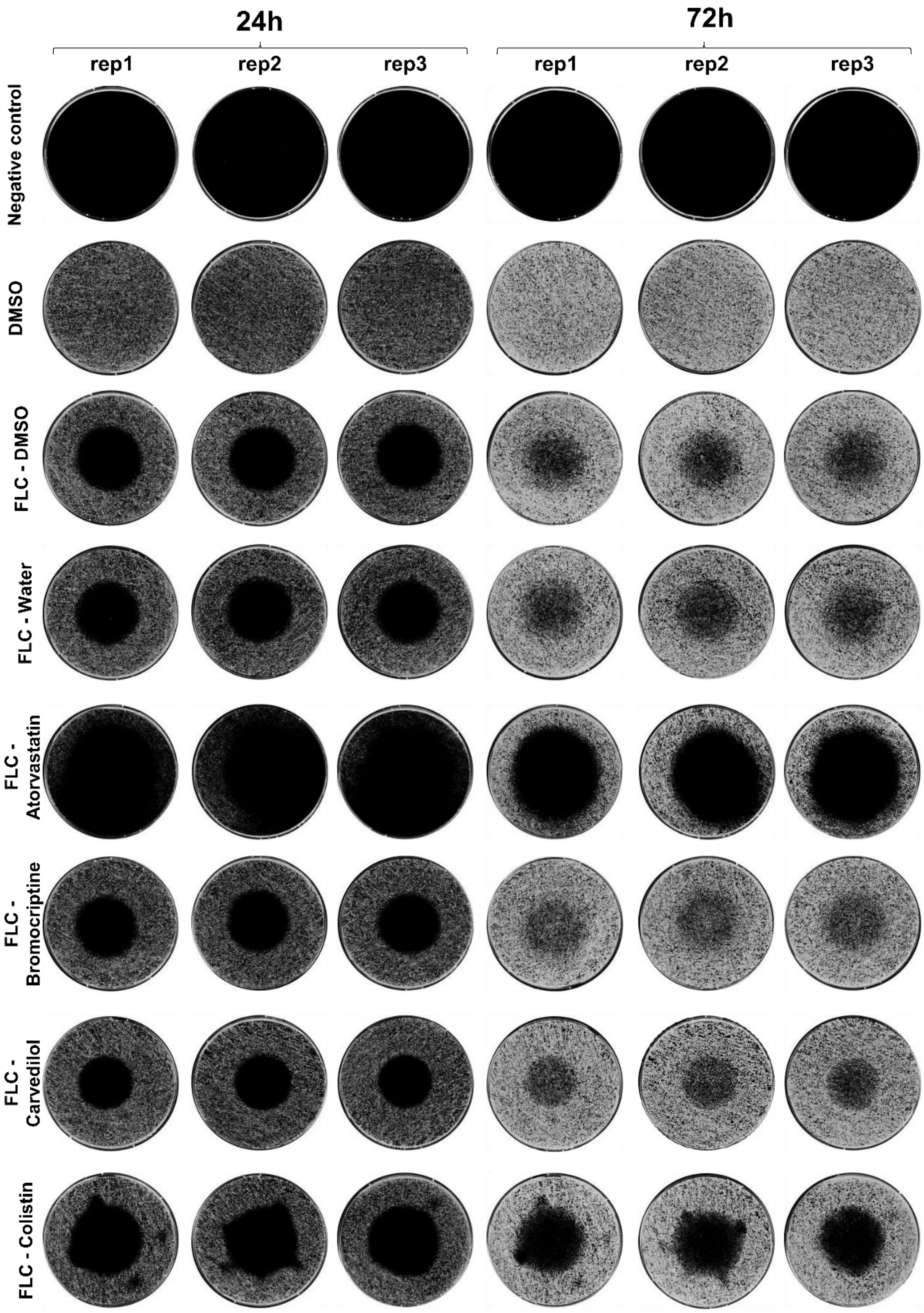

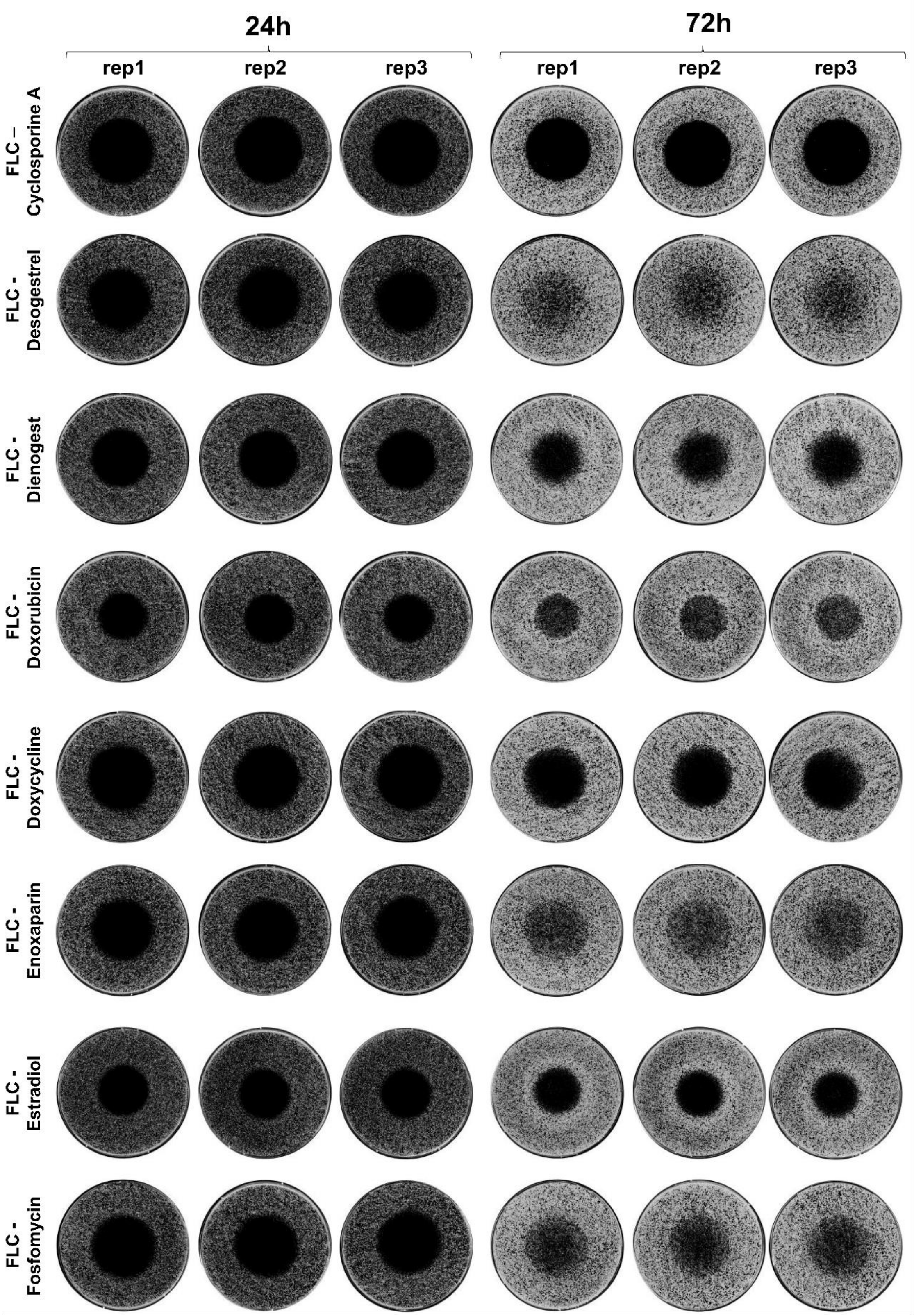
24h
72h
rep1
rep2
rep3
rep1
rep2
rep3
FLC – Cyclosporine A
FLC - Desogestrel
FLC - Dienogest
FLC - Doxorubicin
FLC - Doxycycline
FLC - Enoxaparin
FLC - Estradiol
FLC - Fosfomycin

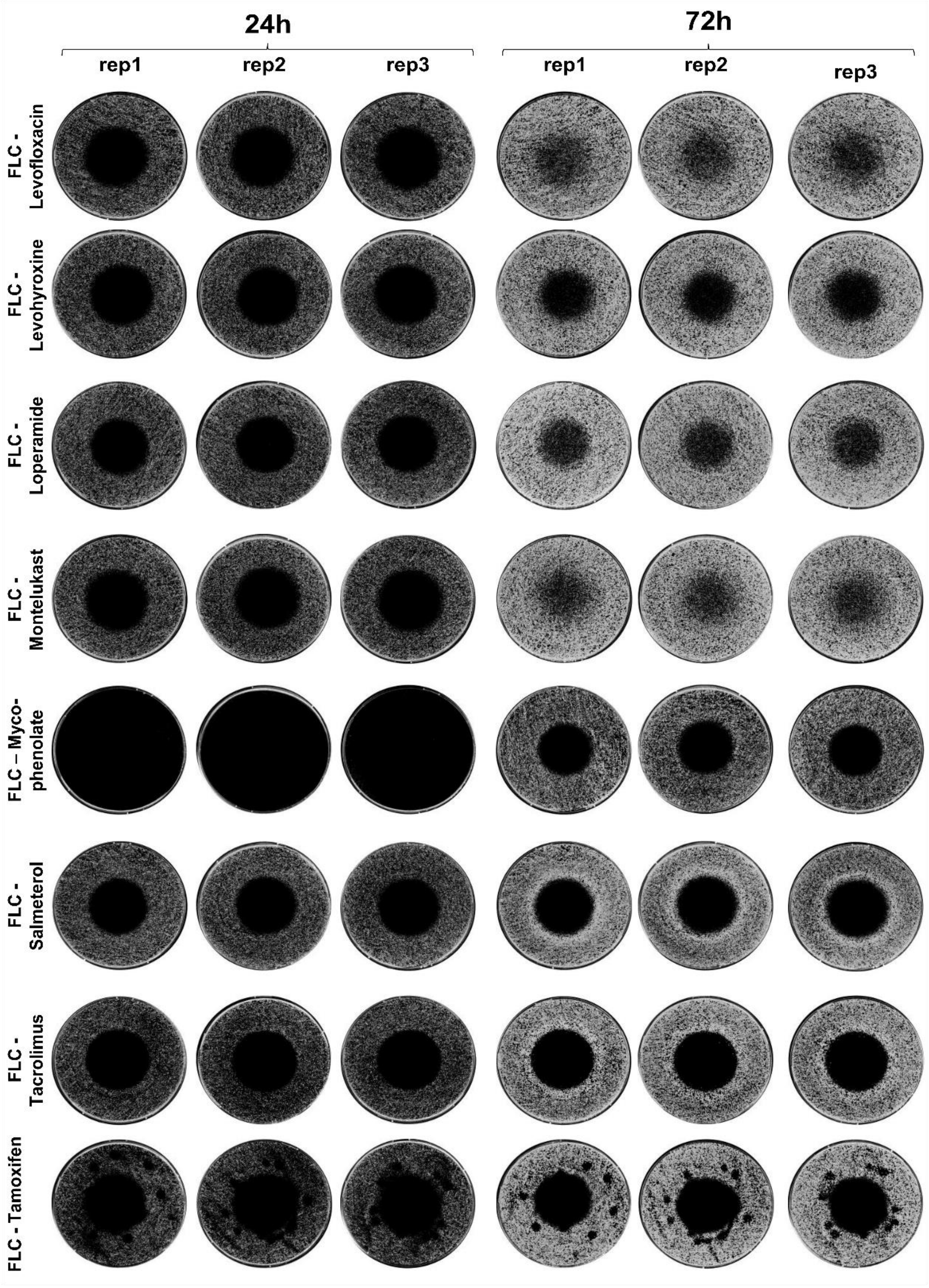
24h
72h
rep1
rep2
rep3
rep1
rep2
rep3
FLC - Levofloxacin
FLC - Levohyroxine
FLC - Loperamide
FLC - Montelukast
FLC – Myco-phenolate
FLC - Salmeterol
FLC - Tacrolimus
FLC - Tamoxifen

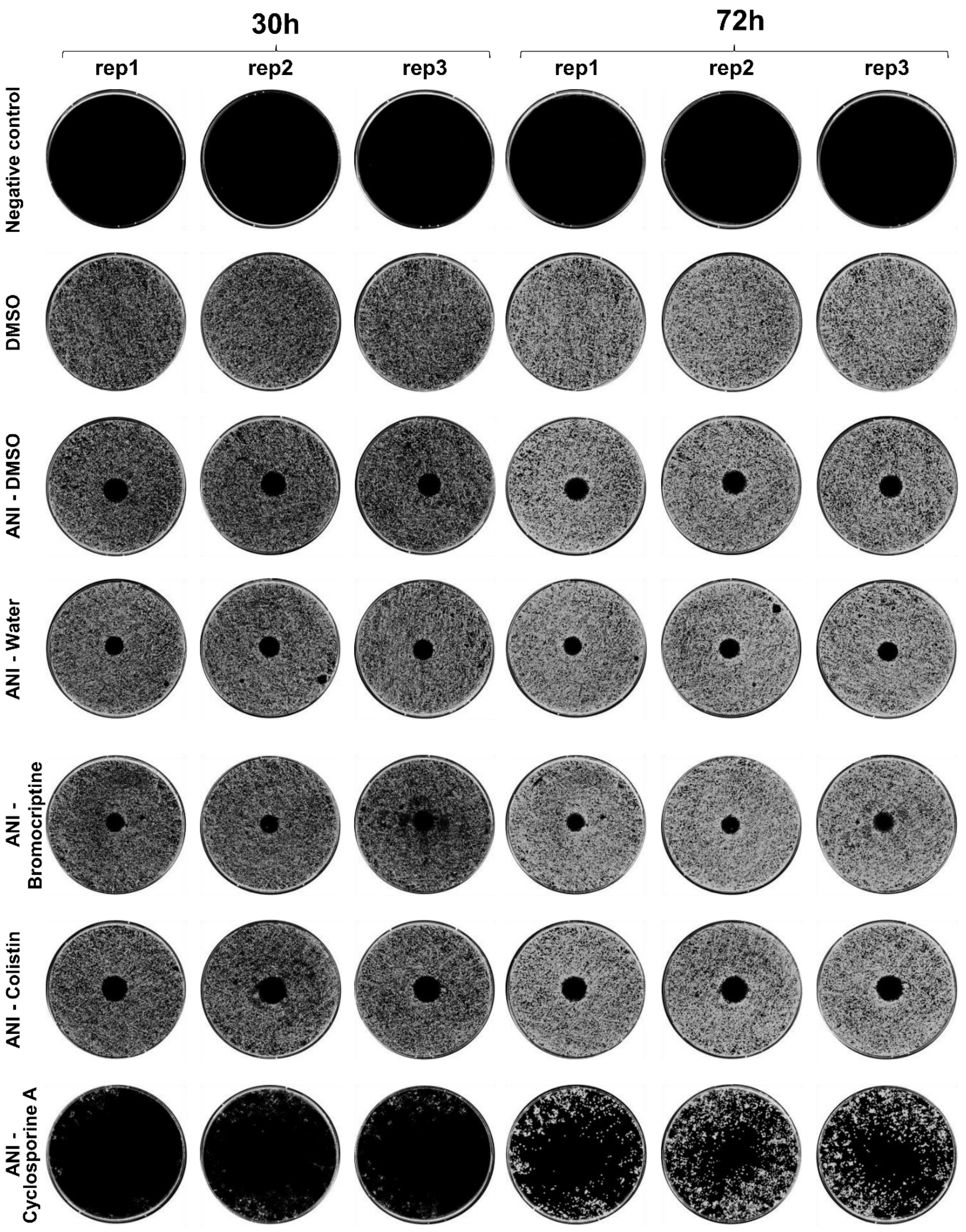
30h
72h
rep1
rep2
rep3
rep1
rep2
rep3
Negative control
DMSO
ANI - DMSO
ANI - Water
ANI - Bromocriptine
ANI - Colistin
ANI - Cyclosporine A

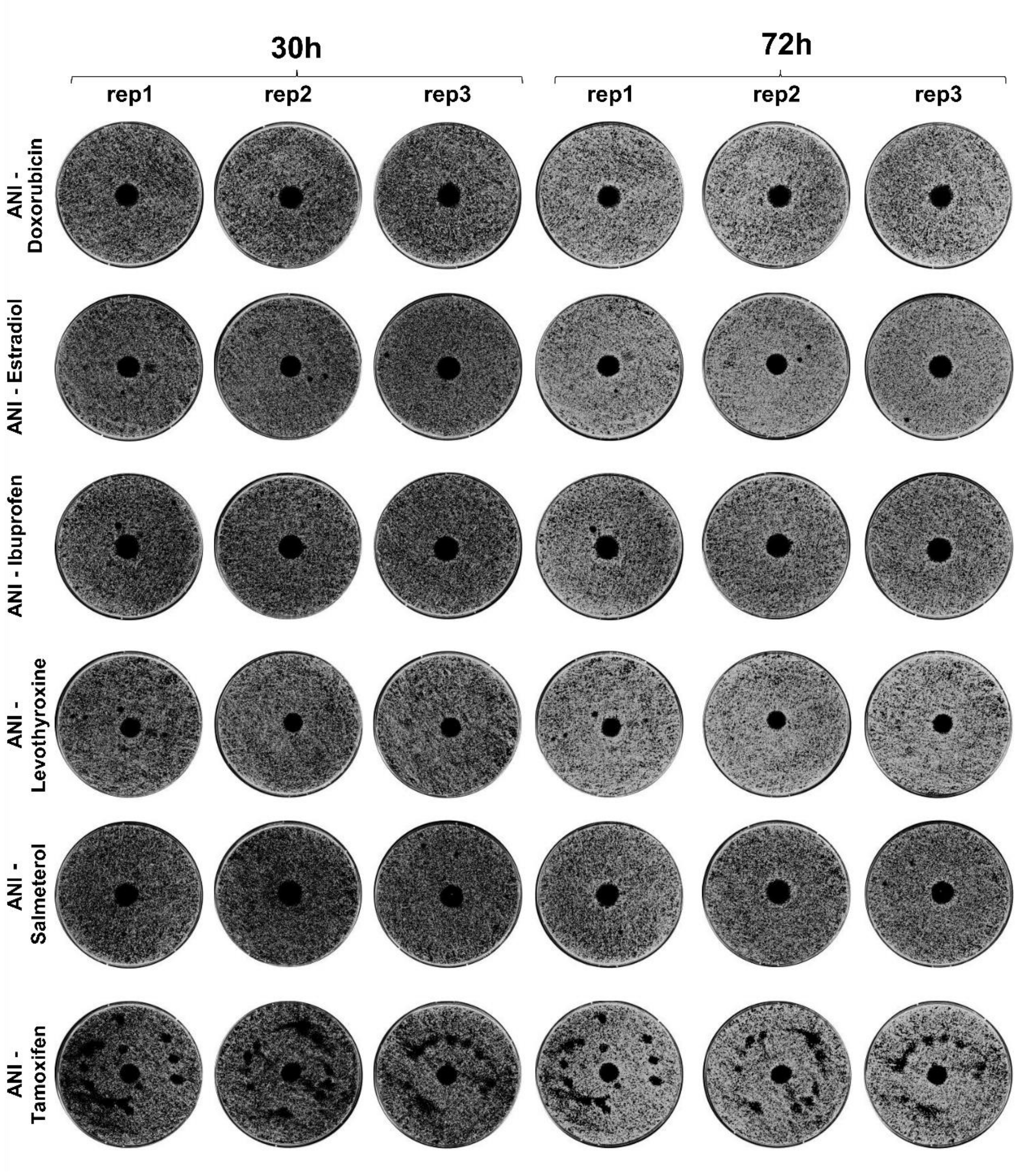
30h
72h
rep1
rep2
rep3
rep1
rep2
rep3
ANI - Doxorubicin
ANI - Estradiol
ANI - Ibuprofen
ANI - Levothyroxine
ANI - Salmeterol
ANI - Tamoxifen

Supplementary Figure 6: Human drugs alter FLC (blue) and ANI (red) resistance and tolerance, measured in DDAs.

(a) FLC resistance were evaluated by measuring the radius of the zone of inhibition (ZOI) after 24 h.

(b) FLC tolerance were determined by measuring the inhibition effect inside the ZOI after 72 h.

(c) ANI resistance measured by the radius of the ZOI after 30 h.

N=3 biological replicates (except for DMSO+FLC, where N=6 biological replicates).

"_w" indicates drugs that were dissolved in water rather than DMSO.

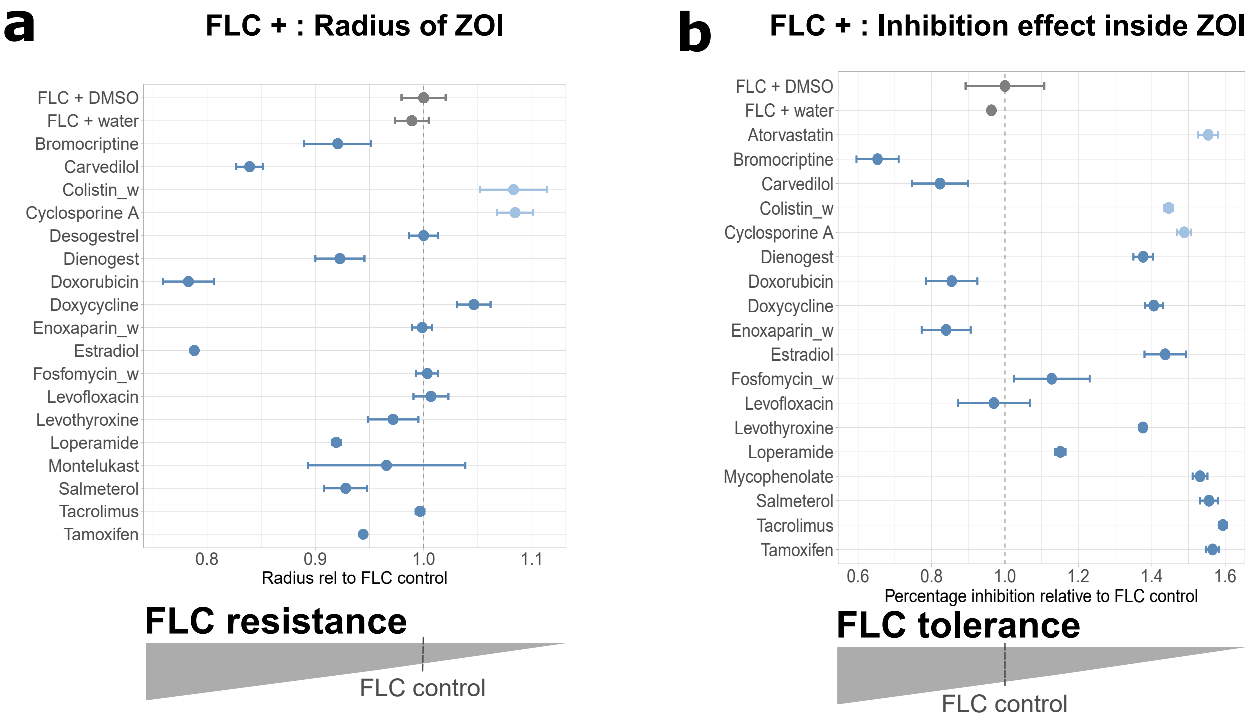

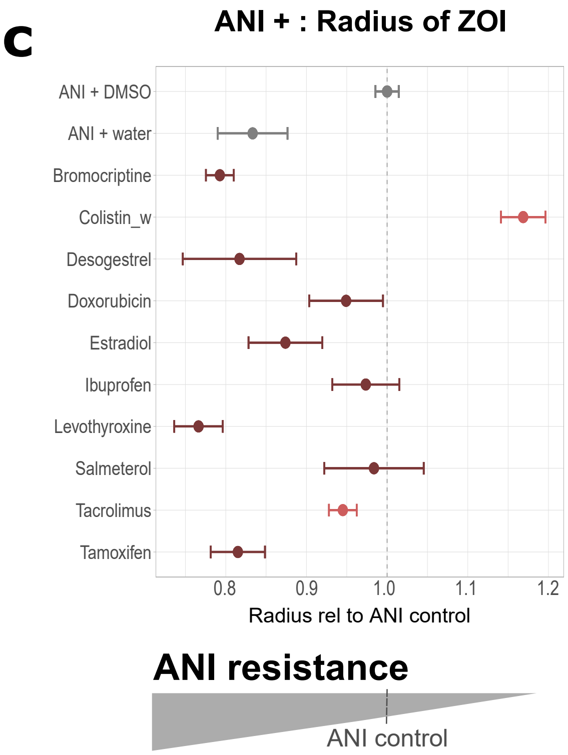

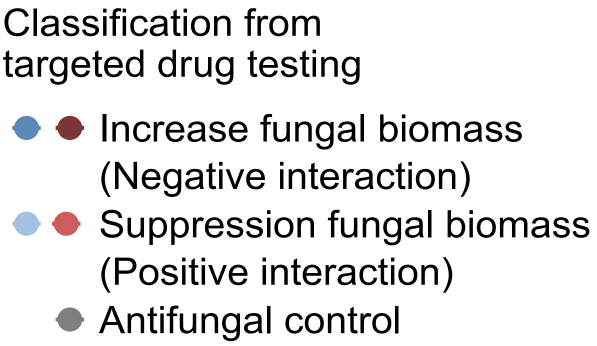

Supplementary Figure7 :

In-vivo tests of FLC antagonists in *G. mellonella* larvae. *G. mellonella* were infected with *C. albicans* and treated with FLC alone, FLC + FLC antagonist, FLC antagonist only or not treated. **(A)** Time of survival up to 50% death of larvae show that most proposed FLC antagonists also shortened the time of *G. mellonella* median survival in most drugs. **(B)** Kaplan-Meier survival analysis of different tested drugs. Compounds which differed significantly from the FLC treatment only are shown in Figure 5. A Wilcoxon rank sum test and a Bonferroni-correction was performed to test statistical differences between the groups, indicated as different letters above the graphs. N=15, 2 replicates per experiment. Right-censored data are depicted as crosses.

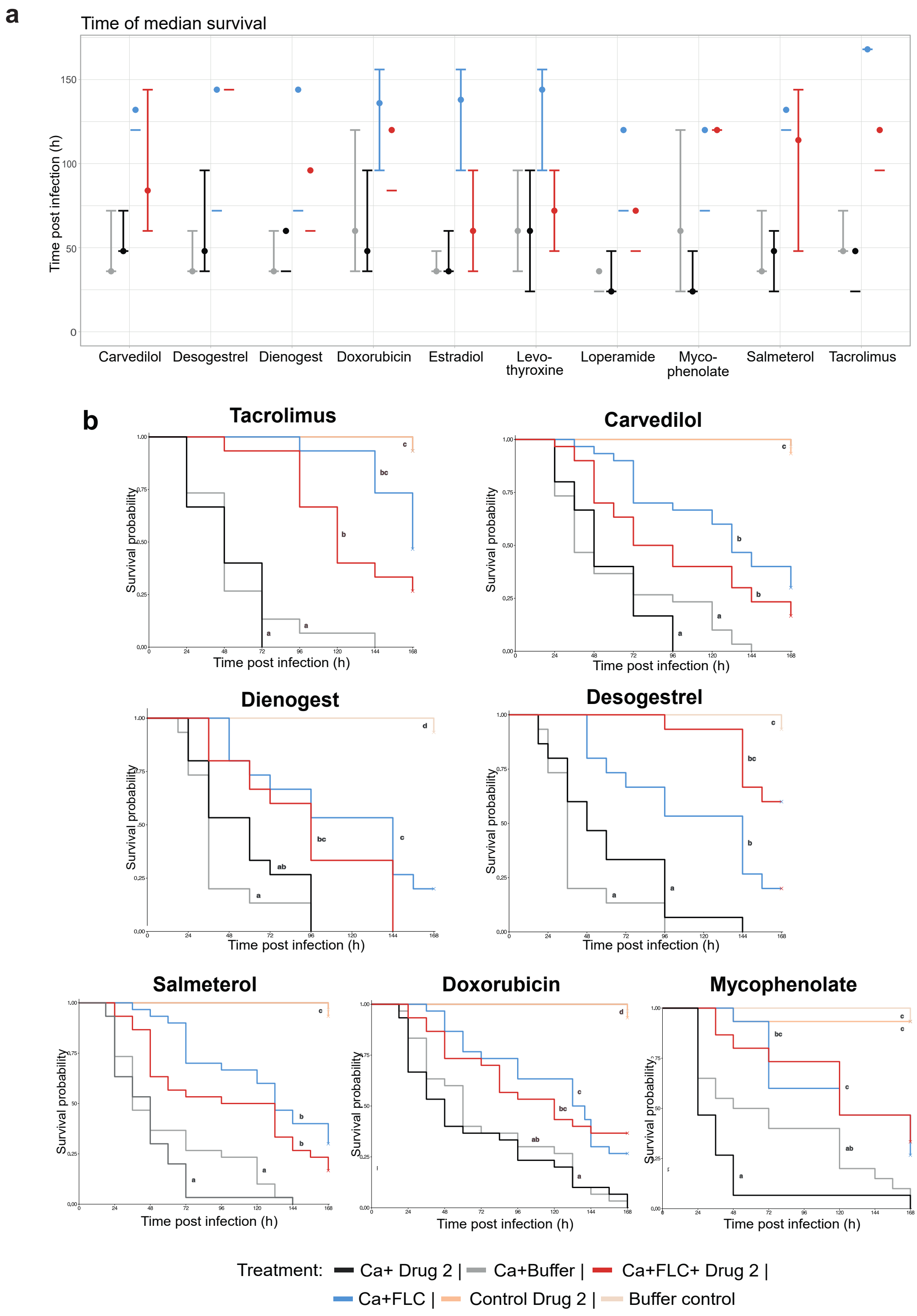

Supplement: Uncited Supplementary Material 1. [file jmm-74-02046-s001.pdf]
